# Recalibrating single-study effect sizes using hierarchical Bayesian models

**DOI:** 10.3389/fnimg.2023.1138193

**Published:** 2023-12-21

**Authors:** Zhipeng Cao, Matthew McCabe, Peter Callas, Renata B. Cupertino, Jonatan Ottino-González, Alistair Murphy, Devarshi Pancholi, Nathan Schwab, Orr Catherine, Kent Hutchison, Janna Cousijn, Alain Dagher, John J. Foxe, Anna E. Goudriaan, Robert Hester, Chiang-Shan R. Li, Wesley K. Thompson, Angelica M. Morales, Edythe D. London, Valentina Lorenzetti, Maartje Luijten, Rocio Martin-Santos, Reza Momenan, Martin P. Paulus, Lianne Schmaal, Rajita Sinha, Nadia Solowij, Dan J. Stein, Elliot A. Stein, Anne Uhlmann, Ruth J. van Holst, Dick J. Veltman, Reinout W. Wiers, Murat Yücel, Sheng Zhang, Patricia Conrod, Scott Mackey, Hugh Garavan, The ENIGMA Addiction Working Group

**Affiliations:** ^1^Shanghai Xuhui Mental Health Center, Shanghai, China; ^2^Department of Psychiatry, University of Vermont College of Medicine, Burlington, VT, United States; ^3^Department of Mathematics and Statistics, University of Vermont College of Engineering and Mathematical Sciences, Burlington, VT, United States; ^4^Department of Psychological Sciences, School of Health Sciences, Swinburne University, Melbourne, VIC, Australia; ^5^Department of Psychology and Neuroscience, University of Colorado Boulder, Boulder, CO, United States; ^6^Department of Psychology, Education and Child Studies, Erasmus University Rotterdam, Rotterdam, Netherlands; ^7^Department of Neurology and Neurosurgery, Montreal Neurological Institute, McGill University, Montreal, QC, Canada; ^8^Department of Neuroscience, The Ernest J. Del Monte Institute for Neuroscience, University of Rochester School of Medicine and Dentistry, Rochester, NY, United States; ^9^Department of Psychiatry, Amsterdam UMC, University of Amsterdam, Amsterdam, Netherlands; ^10^Melbourne School of Psychological Sciences, University of Melbourne, Melbourne, VIC, Australia; ^11^Department of Psychiatry, Yale University School of Medicine, New Haven, CT, United States; ^12^Laureate Institute for Brain Research, Tulsa, OK, United States; ^13^Department of Psychiatry at Oregon Health and Science University, Portland, OR, United States; ^14^David Geffen School of Medicine, University of California at Los Angeles, Los Angeles, CA, United States; ^15^Neuroscience of Addiction and Mental Health Program, Healthy Brain and Mind Research Centre, School of Behavioural & Health Sciences, Faculty of Health Sciences, Australian Catholic University, Australia; ^16^Behavioural Science Institute, Radboud University, Nijmegen, Netherlands; ^17^Department of Psychiatry and Psychology, University of Barcelona, Barcelona, Spain; ^18^Clinical NeuroImaging Research Core, Division of Intramural Clinical and Biological Research, National Institute on Alcohol Abuse and Alcoholism, Bethesda, MD, United States; ^19^VA San Diego Healthcare System and Department of Psychiatry, University of California San Diego, La Jolla, CA, United States; ^20^Orygen, Parkville, VIC, Australia; ^21^Centre for Youth Mental Health, The University of Melbourne, Melbourne, VIC, Australia; ^22^School of Psychology and Illawarra Health and Medical Research Institute, University of Wollongong, Wollongong, NSW, Australia; ^23^SA MRC Unit on Risk and Resilience in Mental Disorders, Department of Psychiatry and Neuroscience Institute, University of Cape Town, Cape Town, South Africa; ^24^Neuroimaging Research Branch, Intramural Research Program, National Institute on Drug Abuse, Baltimore, MD, United States; ^25^Department of Child and Adolescent Psychiatry and Psychotherapy, Technische Universität Dresden, Dresden, Germany; ^26^Addiction Development and Psychopathology (ADAPT)-Lab, Department of Psychology and Center for Urban Mental Health, University of Amsterdam, Amsterdam, Netherlands; ^27^BrainPark, Turner Institute for Brain and Mental Health, School of Psychological Sciences, and Monash Biomedical Imaging Facility, Monash University, Melbourne, VIC, Australia; ^28^Department of Psychiatry, Université de Montreal, CHU Ste Justine Hospital, Montreal, QC, Canada

**Keywords:** effect size recalibration, hierarchical Bayesian model, case-control differences, substance dependence, small sample size, inflated effect size

## Abstract

**Introduction:**

There are growing concerns about commonly inflated effect sizes in small neuroimaging studies, yet no study has addressed recalibrating effect size estimates for small samples. To tackle this issue, we propose a hierarchical Bayesian model to adjust the magnitude of single-study effect sizes while incorporating a tailored estimation of sampling variance.

**Methods:**

We estimated the effect sizes of case-control differences on brain structural features between individuals who were dependent on alcohol, nicotine, cocaine, methamphetamine, or cannabis and non-dependent participants for 21 individual studies (Total cases: 903; Total controls: 996). Then, the study-specific effect sizes were modeled using a hierarchical Bayesian approach in which the parameters of the study-specific effect size distributions were sampled from a higher-order overarching distribution. The posterior distribution of the overarching and study-specific parameters was approximated using the Gibbs sampling method.

**Results:**

The results showed shrinkage of the posterior distribution of the study-specific estimates toward the overarching estimates given the original effect sizes observed in individual studies. Differences between the original effect sizes (i.e., Cohen's d) and the point estimate of the posterior distribution ranged from 0 to 0.97. The magnitude of adjustment was negatively correlated with the sample size (r = −0.27, *p* < 0.001) and positively correlated with empirically estimated sampling variance (r = 0.40, *p* < 0.001), suggesting studies with smaller samples and larger sampling variance tended to have greater adjustments.

**Discussion:**

Our findings demonstrate the utility of the hierarchical Bayesian model in recalibrating single-study effect sizes using information from similar studies. This suggests that Bayesian utilization of existing knowledge can be an effective alternative approach to improve the effect size estimation in individual studies, particularly for those with smaller samples.

## Introduction

Neuroimaging is a primary tool to study neural phenotypes of human health and disease. However, neuroimaging studies are often conducted on small samples (Poldrack et al., 2017; Turner et al., 2018; Szucs and Ioannidis, 2020). For example, the median participant numbers in groups were 24 for the 163 most-cited clinical MRI studies between 1990 and 2012 (Szucs and Ioannidis, 2020). Coupled with growing concerns about inflated effect sizes and low reproducibility in neuroimaging studies with small samples (Button et al., 2013; Poldrack et al., 2017; Turner et al., 2018; Marek et al., 2022), the field faces a crisis of relevance if published studies cannot be replicated.

Obtaining accurate (reproducible) effect sizes is essential to establishing a reliable empirical database of neuroimaging findings. Multisite large-scale neuroimaging consortia, such as the Enhancing Neuroimaging Genetics through Meta-Analysis (ENIGMA) project and the Adolescent Brain Cognitive Development (ABCD) study, have been established to address concerns over the rigor and reproducibility of many neuroimaging and genomic findings. The ENIGMA Addiction working group leverages the statistical power of the combined yield of existing datasets pooled using the ENIGMA protocols to examine the neural and genetic bases of addiction (Mackey et al., 2016). The ABCD project is generating a comprehensive dataset on almost 12,000 adolescents with neuroimaging data obtained every 2 years over 10 years (Casey et al., 2018; Garavan et al., 2018). The availability of these large samples has facilitated a shift in analytic focus away from statistical significance testing toward the potentially more informative comparison of effect sizes (Etkin, 2019).

Empirically determined effect sizes from large-scale neuroimaging studies are smaller than expected by traditional standards (Owens et al., 2021; Marek et al., 2022). A previous study based on ABCD data (*N* = 11,878) revealed that the largest observed univariate correlation between behavioral phenotypes (e.g., cognition and mental health) and brain structure/function was 0.14 (Marek et al., 2022). Owens and colleagues further calculated the Pearson's correlation among hundreds of questionnaire and task measures from the ABCD study and showed that the median in-sample correlation was 0.03 (Owens et al., 2021). A large-scale, case-control comparison study by the ENIGMA Addiction working group revealed smaller volume or cortical thickness in addiction samples (*N* = 2,140) compared with healthy controls (*N* = 1,100), with the largest Cohen's *d* effect size of −0.087 observed in the left hippocampus (Mackey et al., 2019). A separate analysis showed that the largest observed Cohen's *d* effect size of substance dependence in structural asymmetries was 0.15 in the nucleus accumbens (Cao et al., 2021). These findings not only underscore the importance of large samples for detecting subtle effects but also should trigger a recalibration in researchers' expectations of the true effect sizes in neuroimaging studies. No study has yet addressed how effect sizes in neuroimaging studies with small samples could be adjusted on the basis of a pooled database of already completed studies.

Here, we used a collection of 21 separate structural brain MRI studies from the ENIGMA Addiction Working Group with data from individuals who were dependent on alcohol, nicotine, cocaine, methamphetamine, or cannabis (*n* = 903) and non-dependent participants (*n* = 996). The effect sizes of case-control differences in brain structural features were estimated using Cohen's *d* for each study and then modeled using a hierarchical Bayesian approach. In a typical hierarchical Bayesian model, low-level parameters (e.g., parameters for a study-specific distribution) are sampled from a higher-level parameter distribution (e.g., the overarching distribution of the study-specific parameters). The estimated study-specific sampling variance was incorporated into the hierarchical model to modulate the estimation of study-specific parameters. As a property of the hierarchical Bayesian model, we expected the shrinkage of the posterior distribution of the study-specific estimates toward the overarching estimates based on the original effect sizes observed in individual studies. In addition, we anticipated that smaller studies would have a larger estimated sampling variance. Consequently, when the point estimate of the study-specific posterior distribution was used as the Bayesian adjusted effect size, greater adjustments from the original effect sizes to the Bayesian adjusted effect sizes would be observed in smaller studies than in larger studies.

## Methods

### Behavioral phenotyping

Data were contributed from 27 laboratories on 3,046 individuals, including 1,932 who were diagnosed with current dependence on at least one of the five substances of interest: alcohol, nicotine, cocaine, methamphetamine, and cannabis. The data used in the present study was a subset of data described previously (Mackey et al., 2019; Cao et al., 2021). Individuals were excluded if they had a lifetime history of neurological diseases, a current DSM-IV axis I diagnosis other than depressive and anxiety disorders, or any contraindication for MRI. Non-dependent participants may have used psychoactive substances recreationally but did not meet DSM-IV criteria for substance dependence. After the quality control steps described below, 2,792 participants remained, including 1,792 participants with dependence. Then, six studies that had only dependent or non-dependent participants were excluded, resulting in 21 studies with 1,899 participants including 903 participants with dependence included in the present analysis. Study-specific summary demographic statistics for these participants are provided in Supplementary Table 1.

### Preparation of structural MRI data

The volumes of seven bilateral subcortical regions and thicknesses and surface areas of 34 bilateral cortical regions from both hemispheres were extracted from structural T1-weighted MRI brain scans using *FreeSurfer* (version 5.3) (18). A standardized protocol of quality control procedures was performed at each site (http://enigma.ini.usc.edu/protocols/imaging-protocols/), which includes detection of outliers and visual inspection of all data in a series of standard planes. An additional visual inspection was performed at the University of Vermont on a randomly selected subsample of participants to ensure uniformity of quality control across sites. Scanner and acquisition details at each site have been published (Mackey et al., 2019; Cao et al., 2021).

### Data harmonization

To address the potential differences between sites, a harmonization technique ComBat, was applied to remove unwanted study effects while preserving between-subject biological variability (i.e., diagnosis of dependence, age, and sex; Fortin et al., 2017, 2018; Radua et al., 2020). ComBat was originally proposed for gene expression microarray data (Johnson et al., 2007), and proved to be effective in neuroimaging studies (Fortin et al., 2017, 2018; Radua et al., 2020). The study-harmonized data were used to estimate the study-specific sampling variance while considering the sample profiles as described below. We have performed a sensitivity analysis using unharmonized data to explore the impact of ComBat on the adjusted effect sizes. To simplify the analysis, these sensitivity analyses were only performed on regional CT. As shown in Supplementary Figures 6, 7, analyses using non-ComBat-adjusted data revealed no substantial differences compared to the main results with ComBat harmonization, suggesting the application of ComBat had inconsequential effects on both overarching and study-specific effect size estimations.

### Effect size estimation

For each study, the association between substance use and the ROI-level structural measure was modeled by a series of linear regressions. Diagnosis (dependent vs. non-dependent), age, sex (male vs. females), and ICV were included as predictors. An effect size of diagnosis was calculated for each ROI and each site using the following formula (Rosenthal et al., 1994):


$$\begin{array}{l} Cohen^{\prime }s\;d=\frac{t\times \left(n1+n2\right)}{\sqrt{\left(n1\times n2\right)}\;\times \sqrt{df}} \end{array}$$

Where *n*1 and *n*2 represent the numbers of cases and controls, respectively, *t* is the test statistic associated with diagnosis and *df* is degrees of freedom. The Cohen's *d* effect sizes in each study were included as the observations in the following hierarchical Bayesian model. As our primary aim was to showcase the effectiveness of hierarchical Bayesian models in effect size calibration, we did not include interaction terms in the case-control comparison models. This approach is consistent with our previous studies (Mackey et al., 2019; Cao et al., 2021, 2023) as well as with the models used in studies from other ENIGMA working groups (Schmaal et al., 2017; Boedhoe et al., 2018; Van Erp et al., 2018; Whelan et al., 2018).

### Hierarchical Bayesian model

Bayesian inference tempers observed effects on the basis of prior expectations (Kruschke, 2014). In a typical hierarchical Bayesian model, low-level parameters (e.g., parameters for a study-specific distribution of effect size) are sampled from a higher-level parameter distribution (e.g., the overarching distribution of the study-specific parameters). Adjusting low-level parameters toward the overarching parameters is referred to as shrinkage of the parameter.

**Figure 1:**
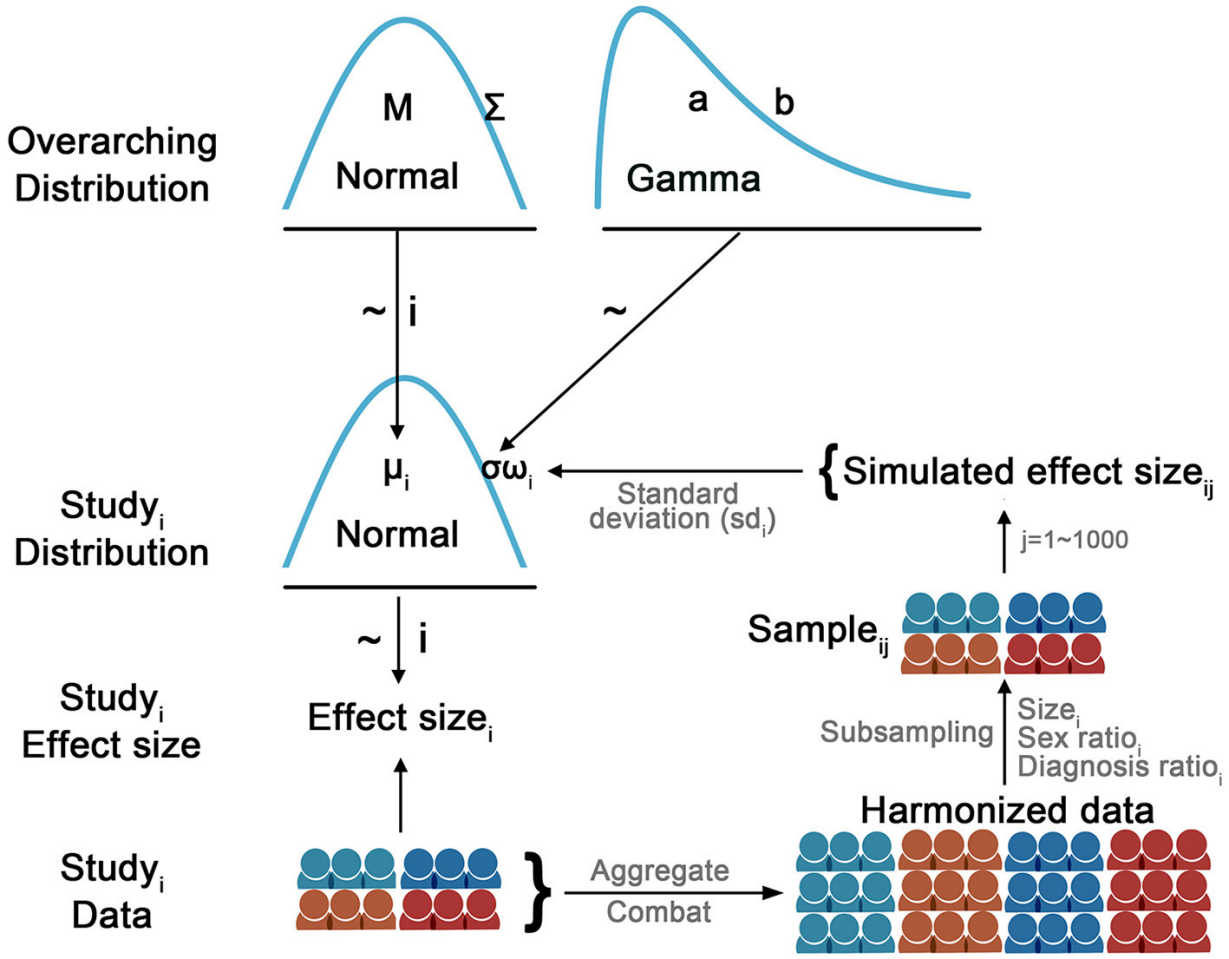
Diagram illustrates the hierarchical Bayesian model used to model the study-specific effect size. The observed effect size for the *i*^*th*^ study was sampled from a study-specific normal distribution *N*(μ_*i*_, ω_*i*_σ). The study-specific μ_*i*_ was assumed to be sampled from a higher-order normal distribution *N*(*M*, Σ). The common part of the variance of the study-specific distribution σ was sampled from a higher-order *Gamma*(*a, b*) distribution and weighted by the study-specific sampling variance ω_*i*_. The study-specific sampling variance ω_*i*_ for the *i*^*th*^ study was estimated as follows: a sample with the same sample size and the same ratios of diagnosis and sex was drawn from the harmonized data. Then, a linear regression was performed on the drawn sample and the Cohen's *d* effect size was calculated. After repeating this procedure 1,000 times, a distribution of 1,000 simulated effect sizes based on the same diagnosis and sex ratio was created for the *i*^*th*^ study. The study-specific sampling variance ω_*i*_ was calculated as the standard deviation of the simulated effect sizes, which was incorporated into the study-specific model. This strategy allowed the model to accommodate differences in sample size as well as the potential impact of sample profiles (e.g., sample size, diagnosis, and sex ratios) on the sampling variance when estimating the study-specific parameters. That is, if an individual study had a low estimated sampling variance, it would have a small weight (ω) on the common part of the variance (σ) in the study-specific distribution. Per JAGS convention, the precision of the distribution (i.e., the reciprocal of the variance: 1/σ or 1/ε) was modeled in the JAGS.

A hierarchical Bayesian model was used to model the overarching and study-specific distribution of effect sizes for substance dependence associations with cortical thickness, cortical surface area and subcortical volumes. As shown in Figure 1, the observed effect size for the *i*^*th*^ study was sampled from a study-specific normal distribution *N*(μ_*i*_, ω_*i*_σ). The study-specific μ_*i*_ was assumed to be sampled from a higher-order normal distribution *N*(*M*, Σ). The common part of the variance of the study-specific distribution σ was sampled from a higher-order *Gamma*(*a, b*) distribution and weighted by the study-specific sampling variance ω_*i*_. The study-specific sampling variance ω_i_ for the *i*^*th*^ study was estimated as follows: a sample with the same sample size and the same ratios of diagnosis and sex was drawn from the harmonized data. Then, a linear regression was performed on the drawn sample and the Cohen's *d* effect size was calculated. After repeating this procedure 1,000 times, a distribution of 1,000 simulated effect sizes based on the same diagnosis and sex ratio was created for the *i*^*th*^ study. The study-specific sampling variance ω_*i*_ was calculated as the standard deviation of the simulated effect sizes, which was incorporated into the study-specific model. This strategy allowed the model to accommodate differences in sample size as well as the potential impact of sample profiles (e.g., sample size, diagnosis, and sex ratios) on the sampling variance when estimating the study-specific parameters. That is, if an individual study had a low estimated sampling variance, it would have a small weight (ω) on the common part of the variance (σ) in the study-specific distribution.

Gibbs sampling, a Markov chain Monte Carlo (MCMC) algorithm, was employed to approximate the posterior distribution of parameters of interest (i.e., μ and *M*) conditioned on the observed data. JAGS along with R packages *coda* and *rjags* were used to implement the Gibbs sampling (Plummer, 2003, 2016; Plummer et al., 2006). Mild informative prior distributions were set for the *M*, σ and Σ parameters. Specifically, *M* was sampled from a prior distribution of *N*(0,10), and σ and Σ were sampled from a *Gamma* distribution with a mode of 1 and standard deviation of 10 (Kruschke, 2014). Per JAGS convention, the precision of the distribution (i.e., the reciprocal of the variance: 1/σ or 1/ε) was modeled in the JAGS. Four sampling chains with random initial values were generated based on 100,000 iterations for the parameters. Gelman-Rubin statistic was used to examine the representativeness of the MCMC sampling, with a value of 1 indicating the chains were fully converged. Effective sample size (ESS) was estimated to assess the stability and accuracy of the sampling chains. For each parameter of interest, a minimum ESS of 10,000 was obtained as recommended previously (Kruschke, 2014).

To justify the assumption that the study-specific distributions of effect sizes were normal, we simulated 1,000 effect sizes for each regional measurement by performing case-control comparisons with the same number of participants, maintaining the same sex and diagnostic ratios from the ComBat-harmonized datasets. We then applied the Kolmogorov-Smirnov (KS) test to assess the normality of the simulated effect sizes for each region (Lilliefors, 1967). A *p*-value of < 0.05 indicated statistically significant evidence to reject the null hypothesis (i.e., the simulated effect sizes were drawn from a normal distribution), suggesting that the distribution of effect sizes deviated from normality. As shown in Supplementary Figure 5, only one out of 150 × 21 = 3,150 data points showed an uncorrected *p*-value < 0.05. Therefore, it was appropriate to assume that the study-specific distribution of effect sizes was normal.

Additional sensitivity analyses were performed to explore the potential impact of the choices of Gamma priors on the results using two extreme Gamma priors: a less informative prior with a mode of 1 and an SD of 100, and a more informative prior with a mode of 1 and an SD of 0.1. The distributions for these Gamma priors are shown in Supplementary Figure 8. To simplify the analysis, the sensitivity was only performed on regional CT. As shown in Supplementary results, the sensitivity analyses with two extreme Gamma priors revealed the potential impacts of different Gamma priors on results, which highlights the importance of choosing appropriate priors for the variance parameters. In line with previous recommendations (Kruschke, 2014), our study used the mild informative Gamma prior, which we contend was appropriate given the effect sizes of case-control comparison on imaging phenotypes typically ranged from −1 to 1 (Schmaal et al., 2017; Boedhoe et al., 2018; Van Erp et al., 2018; Whelan et al., 2018; Cao et al., 2021, 2023).

To summarize the resulting posterior distributions of the parameters of interest (i.e., μ and *M*), the highest density value (i.e., posterior mode) was derived as the point estimate of the posterior distribution and the 95% highest density interval (HDI) was reported to indicate the 95% credibility interval of the posterior distributions. The posterior mode of the overarching parameter *M* and the study-specific parameter μ represented the estimate of the overall effect size across studies and the study-specific Bayesian adjusted effect size, respectively, given the original effect sizes. To quantify the performance of the posterior mode in recalibrating the effect sizes of individual studies, the distances between the original and Bayesian adjusted effect sizes were calculated. Then, the magnitude of adjustment was tested against a null hypothesis of zero adjustment using one-sided *t*-tests. Pearson's correlation was performed to examine the relations among the magnitude of adjustment, sample size and sampling variance. In the supplementary analysis, we examined the performance of an alternative point estimate (i.e., posterior mean) in recalibrating the effect sizes of individual studies. The *ggplot2, ggseg* (Mowinckel and Vidal-Piñeiro, 2020) and *ggridges* packages were used to visualize results. Computations were performed, in part, on the Vermont Advanced Computing Core. The data that support the findings of this study are available from the ENIGMA Addiction Working Group (https://www.enigmaaddictionconsortium.com/). The code used for the analysis is available on GitHub (https://github.com/zh1peng/paper_code).

## Results

**Figure 2:**
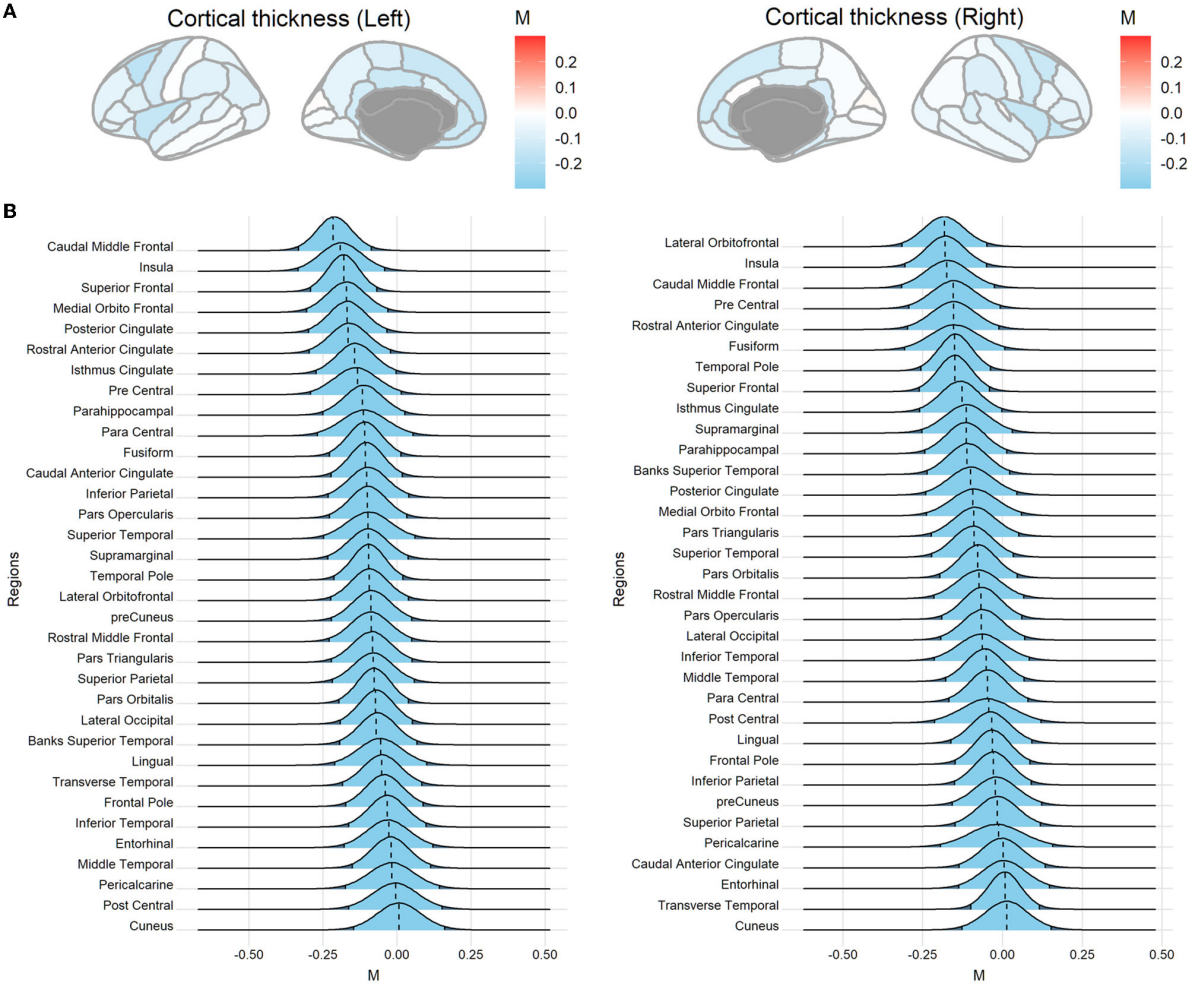
Posterior distributions of the overarching parameter *M* for cortical thickness. **(A)** Posterior mode for each region mapped onto the brain. **(B)** The posterior distribution for cortical regions. The dashed line indicates the posterior mode and the light blue area denotes the 95% highest density interval (HDI). Regions are sorted by the mode value of the distribution.

**Figure 3:**
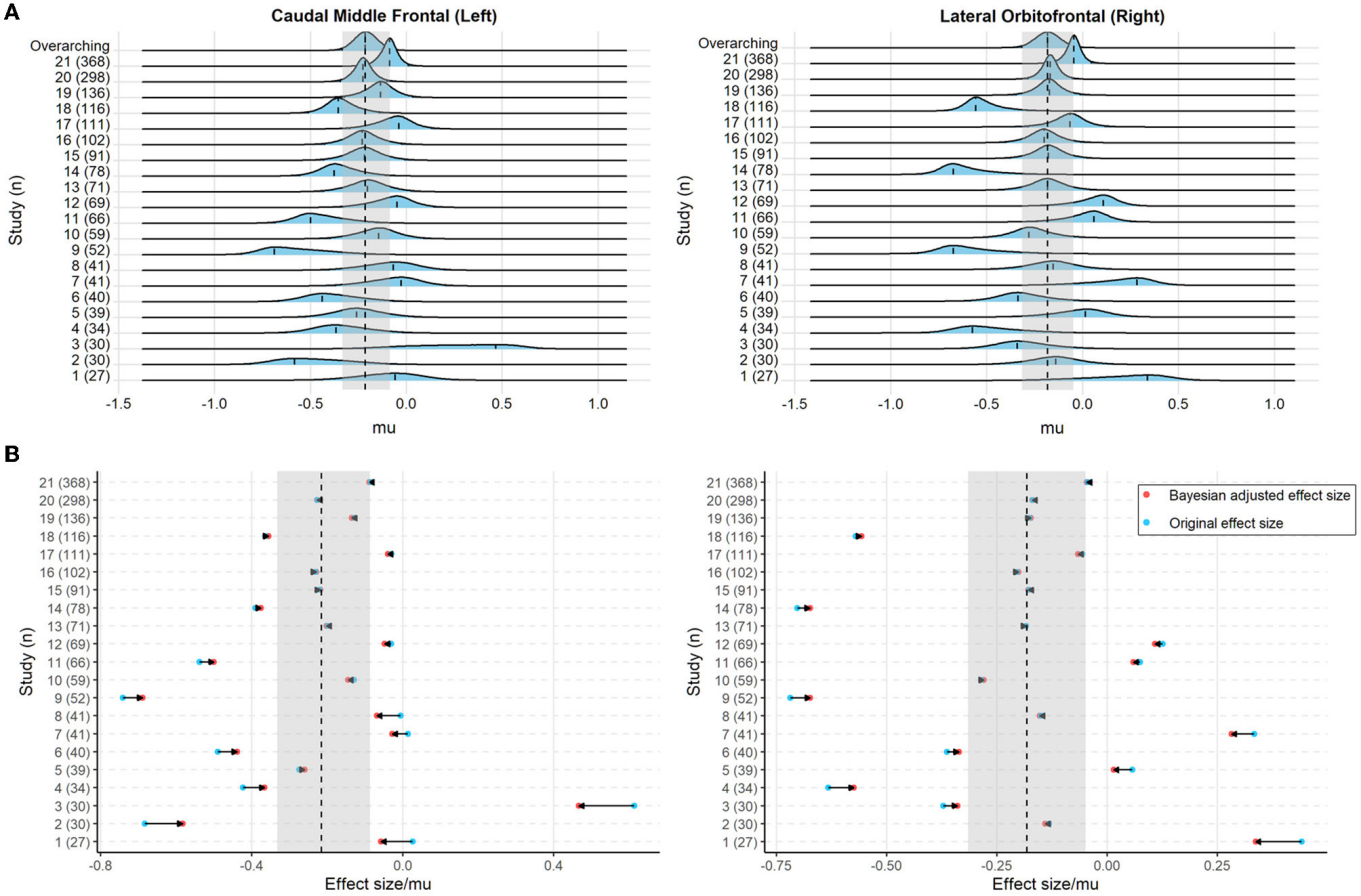
**(A)** The posterior distribution of the study-specific parameter μ and the overarching parameter *M* (top line) for the cortical thickness of the left caudal middle frontal cortex and right lateral orbitofrontal cortex. The dashed line indicates the posterior mode of the parameters and the light blue, as well as the gray shaded area, denotes the 95% highest density interval (HDI). **(B)** The original effect size (blue) and Bayesian adjusted effect size (red; i.e., the posterior mode of the study-specific parameter μ) for the cortical thickness of the left caudal middle frontal cortex and right lateral orbitofrontal cortex. The horizontal arrow indicates the adjustment from the original effect size toward the Bayesian adjusted effect size. The dashed line and the gray shaded area denote the posterior mode and the 95% HDI of the overarching parameter *M*, respectively.

Sample characteristics of individual studies are shown in Supplementary Table 1. Figure 2 shows the posterior mode, and the 95% HDI of the overarching parameter *M* for the regional cortical thickness. Supplementary Figures 1, 2 show the results of the regional surface area and subcortical volume. The descriptive summaries of the posterior distribution are reported in Supplementary Table 2. Most regions had negative posterior mode values, suggesting widespread lower cortical thickness, surface area, and subcortical volume in substance-dependent participants compared to controls. The posterior distribution of the study-specific parameter μ exhibited shrinkage toward the posterior distribution of the overarching parameter *M*. Examples for the left caudal middle frontal cortex and right lateral orbitofrontal cortex that showed largest point estimates of *M* are illustrated in Figure 3. Two additional examples are shown in Supplementary Figure 4. When the posterior mode of parameter μ was used as the Bayesian adjusted estimate of the study-specific effect size, lower Bayesian adjusted effect sizes were found when compared to the original effect sizes (see Supplementary Table 3). The negative correlation (*r* = −0.27, *p* < 0.001) between the magnitude of adjustment and sample size indicated smaller studies tended to have greater adjustments. As expected, smaller studies also showed larger estimated sampling variance across regions, where the study size explained 67% of the variance in the sampling variance across regions. Moreover, the magnitude of the adjustment was positively correlated with the sampling variance (*r* = 0.40, *p* < 0.001), meaning studies with large sampling variance had a greater magnitude of adjustment compared to those with small sampling variance. This proved the effectiveness of incorporating sampling variance in the model.

As shown in Supplementary results, similar results were found when the posterior mean was used as the point estimate of the posterior distribution of the parameter μ. The posterior mean as point estimates for study-specific posterior distribution resulted in more shrinkage toward the overarching distribution across regions and thus led to greater adjustments from the original to the Bayesian adjusted effect sizes when compared to the posterior mode.

## Discussion

In the present study, we proposed a hierarchical Bayesian model to estimate an overarching effect size derived from multiple individual case-control comparison studies and employed it to recalibrate the observed study-specific effect sizes. To demonstrate the effectiveness of the framework, 21 individual studies with varied sample sizes from different collection sites were analyzed. The results indicated that the posterior mode of the overarching parameter *M* was negative across most brain structural features, which is consistent with previous findings suggesting widespread lower cortical thickness, surface area and subcortical volumes in participants with substance dependence compared to non-dependent participants (Mackey et al., 2019). Notably, the posterior mode of the overarching parameter *M* was generally small, with a maximum estimate being −0.244 in the left hippocampus. This supports previous findings based on large-scale data (Mackey et al., 2019; Cao et al., 2021; Owens et al., 2021; Marek et al., 2022). Therefore, the effect sizes in neuroimaging studies may be relatively subtle and require large samples to detect.

For individual studies, smaller studies showed greater sampling variance across brain measures and tended to yield larger original effect sizes. This observation is consistent with previous findings demonstrating the overestimation of effect sizes in small studies (Poldrack et al., 2017). By modeling the study-specific original effect sizes with the hierarchical Bayesian approach, we found that the posterior distribution of the study-specific parameter μ exhibited shrinkage toward that of the overarching parameter *M*. This was mainly attributed to the hierarchical Bayesian model where the estimation of low-level parameters was governed by the overarching parameters. Notably, the hierarchical Bayesian approach has been usefully adopted for random-effects meta-analysis of existing studies to derive overall effects across studies (Röver, 2020). By contrast, we were more interested in the posterior distribution of the study-specific parameter μ, since the point estimate of the posterior distribution (i.e., posterior mode) can be used as the Bayesian adjusted effect size for an individual study. We found that the individually estimated effect sizes could be calibrated by the “peer-effect” in a collection of similar studies. In the supplementary analysis, the posterior mean of the posterior distribution of the study-specific parameter μ was used as the Bayesian adjusted effect size. This alternate approach resulted in greater adjustments in the magnitude of the original effect sizes compared to that of the posterior mode, indicating that the choice of the point estimates (e.g., posterior mean) can impact on the size of the posterior adjustment.

The study-specific sampling variance was incorporated into the hierarchical Bayesian model to modulate the estimation of the study-specific distribution. This strategy was proven effective as the sampling variances were correlated with the magnitude of adjustment from the original effect sizes to the Bayesian adjusted effect sizes. In the present framework, the study-specific sampling variance was estimated by simulating “a similar study” from the study-harmonized datasets while preserving the sex and diagnostic ratios of the specific study. Compared to directly using the sample size as the weight (ω) to modulate the study-specific estimation, the potential impacts of both sample size and the sample profiles (e.g., diagnostic or sex ratio) could be accommodated using the simulated samples. This strategy to utilize large-scale datasets to obtain the tailored sampling variance could be adopted to other publicly available datasets (e.g., UK biobank and ABCD) and extended to other potential sample characteristics of interest (e.g., socioeconomic and ethnicity).

Gratton et al. (2022) have proposed that increasing sample sizes and maximizing effect sizes of interest are two paths toward reliability in brain-behavior association studies (Gratton et al., 2022). As an alternative to improve the reliability of the observed effects in a single study, the Bayesian method described in this work could be used to remedy the effect size estimates that can be inflated in small studies. Similarly, it has been proposed that a large collection of studies that are similar to the study of interest can be used as a default prior (Zwet and Gelman, 2022). The full posterior distribution of the effect sizes from these studies can be used as a prior distribution for new studies. For instance, the posterior distribution for a new study can be directly derived by updating the prior via a closed-form solution (assuming the posterior and prior distribution are conjugated) or approximated using the Gibbs sampling approach by re-running the hierarchical Bayesian model with the observations of new studies.

Another possible implication of the current work is that the posterior of overarching parameter *M* together with the tailored estimation of the sampling variance could be used in a sample size planning analysis. The Bayesian sample size planning framework allows one to incorporate one's goals, desired precision, and belief regarding the sampled population distribution (Kruschke, 2014). There are also a few limitations that may curtail the generalizability of the current work. For instance, we grouped participants as dependent or non-dependent in the current analysis, but the heterogeneity of the participants, type of the substance and co-use of substance were not addressed.

The ComBat harmonization method was applied to minimize non-biological variability between studies that could arise from different imaging protocols, scanners, or other technical factors. However, it should be noted that the harmonization of multisite MRI data is still an active research area (Bayer et al., 2022). Supplementary analysis suggested that the application of ComBat did not substantially impact the adjusted effect sizes. This absence of consequential effects was likely due to the simulated study-specific standard deviation (i.e., the scale factor) having been derived from 1,000 subsampled effect sizes. Repeat sampling may have alleviated any potential effect of non-biological variabilities between studies on the estimation of the study-specific standard deviation. Although not immediately apparent, we contend that the ComBat harmonization is essential to ensure that subsequent subsampling is not confounded by any non-biological variability between studies. This would allow the subsampling and the derived standard deviation to better mimic the effect sizes taken from a single study without between-study confounders. While incorporating ComBat into our proposed framework is appealing and could potentially enhance the model's flexibility, direct integration into a hierarchical Bayesian model may pose methodological challenges and increase complexity. Therefore, in our approach, we utilized the ComBat method as a stand-alone preprocessing step, followed by the estimation of study-specific scale factor based on harmonized data, which ensured optimal performance of both processes within its designated scopes.

Collectively, we demonstrate the utility of hierarchical Bayesian models in recalibrating single-study effect sizes using information obtained from similar studies. Thus, Bayesian utilization of existing knowledge can be an alternative approach to improve the effect size estimation of individual studies.

## Data availability statement

The original contributions presented in the study are included in the article/Supplementary material, further inquiries can be directed to the corresponding author.

## Ethics statement

The studies involving humans were approved by local Ethics Committees or institutional review boards associated with each participating site. The studies were conducted in accordance with the local legislation and institutional requirements. The participants provided their written informed consent to participate in each individual study.

## Author contributions

ZC conceived and performed the analysis, interpreted the results, curated the data, and wrote the manuscript. SM and HG supervised the project, interpreted the results, and edited the manuscript. MM and PCa interpreted the results and edited the manuscript. RC, JO-G, AM, DP, and NSc curated the data and edited the manuscript. WT edited the manuscript. OC, KH, JC, AD, JF, AG, RH, C-SL, AMM, EL, VL, ML, RM-S, RM, MP, LS, RS, NSo, DS, ES, AU, RH, DV, RW, MY, SZ, and PCo contributed to the data acquisition and sharing and edited the manuscript. All authors contributed to the article and approved the submitted version.
