# Supplementary material for: Recalibrating single-study effect sizes using hierarchical Bayesian models
Source: Front Neuroimaging. 2023 Dec 21;2:1138193. doi: 10.3389/fnimg.2023.1138193 (PMC10764546; doi:10.3389/fnimg.2023.1138193)
Supplement: Supplementary file 1 [file Data_Sheet_1.docx]

**Supplementary Materials**

Recalibrating single-study effect sizes using hierarchical Bayesian models

**Contents**

[Supplementary Results 2](#_Toc152939977)

[Supplementary Tables 4](#_Toc152939978)

[Supplementary Figures 12](#_Toc152939979)

[References 24](#_Toc152939980)

## Supplementary Results

When the posterior mean of parameter *µ* was used as the Bayesian adjusted estimates of the study-specific effect size, lower Bayesian adjusted effect sizes were found when compared to the original effect sizes (see **Table S4**). The negative correlation (*r*=-0.35, p<0.001) between the magnitude of adjustment and sample size indicated smaller studies tended to have greater adjustments. Moreover, the magnitude of the adjustment was positively correlated with the sampling variance (*r*=0.48, *p*<0.001), meaning studies with large sampling variance had a greater magnitude of adjustment compared to those with small sampling variance. A paired sample comparison on two types of point estimates (150 regions x 21 studies = 3150 per type) indicated that the posterior mean yielded significantly greater adjustments from the original to Bayesian adjusted effect sizes than that of the posterior mode (*t*=39.454, *df*=3149, *p*<0.001). An example with the left caudal middle frontal cortex and right lateral orbitofrontal cortex is illustrated in **Figure S3**.

The results of the sensitivity analyses with two extreme Gamma priors revealed the potential impacts of different Gamma priors on results. Notably, the precision of the distribution (i.e., the reciprocal of the variance: *1/σ or 1/ Σ*) was modeled in the JAGS. Imposing a more informative Gamma prior (i.e., *Mode* = 1 and *SD* = 0.1) to overarching and study-specific variance parameters (i.e., *Σ* and *σ* respectively) increased the variances of the posterior distribution of the overarching parameter *M* and study-specific parameter *µ* compared to the main results using a mildly informative Gamma prior (see **Figures S9** and **S10**). Due to the large variances present in both overarching and study-specific posterior distributions, the adjustment from the observed effect sizes toward the overarching posterior mode was less pronounced. In contrast, imposing a less informative Gamma prior (i.e., *Mode* = 1 and *SD* = 100) to overarching and study-specific variance parameters (i.e., *Σ* and *σ*  respectively) decreased the variances of the overarching *M* parameter (see **Figure S11**). Moreover, we observed that either the overarching or the study-specific distribution could dominate the hierarchical model (see **Figure S12**). Specifically, in scenarios where the overarching distribution exhibited higher precision over the study-specific distributions (e.g., left caudal middle frontal region), there was a pronounced shrinkage of the study-specific posterior toward the overarching mode. The effect was so substantial that it induced bimodality within the study-specific distributions, suggesting a strong hierarchal influence. On the other hand, when the study-specific estimates demonstrated higher precision (e.g., right lateral orbitofrontal region), they remained unaffected by the overarching distribution. These results highlight the importance of choosing appropriate priors for the variance parameters. In line with previous recommendations (Kruschke, 2014), our study used the mild informative Gamma prior, which we contend was appropriate given the effect sizes of case-control comparison on imaging phenotypes typically ranged from -1 to 1 (Boedhoe, et al., 2018; Cao, et al., 2023; Cao, et al., 2021; Schmaal, et al., 2017; Van Erp, et al., 2018; Whelan, et al., 2018).

## Supplementary Tables

**Table S1.** Sample characteristics of individual studies

| Study Index | Diagnosis | N  (Male%) | Mean Age (standard deviation) | Primary Drug |
| --- | --- | --- | --- | --- |
| 1 | Control | 14 (92.86%) | 16 (1.84) | Cannabis |
|  | Case | 13 (92.31%) | 16.77 (1.01) | Cannabis |
| 2 | Control | 15 (73.33%) | 23.27 (3.67) | Cannabis |
|  | Case | 15 (86.67%) | 22.4 (4.29) | Cannabis |
| 3 | Control | 13 (100%) | 42.85 (8.31) | Cocaine |
|  | Case | 17 (100%) | 40.41 (8.76) | Cocaine |
| 4 | Control | 18 (88.89%) | 35 (10.08) | Cannabis |
|  | Case | 16 (93.75%) | 38.98 (9.24) | Cannabis |
| 5 | Control | 15 (86.67%) | 38.27 (12.65) | Cocaine |
|  | Case | 24 (87.5%) | 35.92 (8.53) | Cocaine |
| 6 | Control | 18 (66.67%) | 46.89 (9.79) | Alcohol |
|  | Case | 22 (68.18%) | 47.64 (10.95) | Alcohol |
| 7 | Control | 30 (63.33%) | 37.13 (10.73) | Methamphetamine |
|  | Case | 11 (72.73%) | 42.82 (10.85) | Methamphetamine |
| 8 | Control | 23 (52.17%) | 19.35 (1.77) | Alcohol |
|  | Case | 18 (66.67%) | 18.72 (1.49) | Alcohol |
| 9 | Control | 24 (100%) | 37.17 (10.68) | Alcohol |
|  | Case | 28 (100%) | 43.43 (9.48) | Alcohol |
| 10 | Control | 29 (100%) | 22.41 (3.31) | Cannabis |
|  | Case | 30 (100%) | 21.03 (2.34) | Cannabis |
| 11 | Control | 45 (46.67%) | 31.56 (9.21) | Cocaine |
|  | Case | 21 (38.1%) | 37.76 (4.58) | Cocaine |
| 12 | Control | 38 (65.79%) | 40.53 (8.26) | Cocaine |
|  | Case | 31 (90.32%) | 40.97 (6.37) | Cocaine |
| 13 | Control | 38 (60.53%) | 22.24 (2.05) | Nicotine |
|  | Case | 33 (72.73%) | 22.79 (3.23) | Nicotine |
| 14 | Control | 40 (62.5%) | 21.85 (2.38) | Cannabis |
|  | Case | 38 (68.42%) | 21.39 (2.42) | Cannabis |
| 15 | Control | 60 (40%) | 19.37 (1.3) | Nicotine |
|  | Case | 31 (29.03%) | 19.74 (1.41) | Nicotine |
| 16 | Control | 37 (48.65%) | 29.89 (11.18) | Cannabis |
|  | Case | 65 (46.15%) | 32.6 (10.92) | Cannabis |
| 17 | Control | 83 (74.7%) | 28.13 (6.7) | Alcohol |
|  | Case | 28 (71.43%) | 37.54 (8.36) | Alcohol |
| 18 | Control | 55 (83.64%) | 26.84 (7.57) | Methamphetamine |
|  | Case | 61 (80.33%) | 27.33 (5.49) | Methamphetamine |
| 19 | Control | 86 (41.86%) | 32.81 (8.53) | Methamphetamine |
|  | Case | 50 (52%) | 32.78 (8.87) | Methamphetamine |
| 20 | Control | 129 (52.71%) | 30.92 (8.27) | Alcohol |
|  | Case | 169 (72.78%) | 38.33 (10.02) | Alcohol |
| 21 | Control | 186 (45.16%) | 29.24 (8.32) | Nicotine |
|  | Case | 182 (57.14%) | 32.3 (9.39) | Nicotine |

**Table S2.** Posterior mode, 95% highest density interval (HDI) and effective sample size (ESS) of the overarching parameter M for subcortical and cortical regions.

|  |  | Left Hemisphere | | | | Right Hemisphere | | | |
| --- | --- | --- | --- | --- | --- | --- | --- | --- | --- |
|  |  |  | 95% HDI | |  |  | 95% HDI | |  |
|  | Regions | M | Lower | Upper | ESS | M | Lower | Upper | ESS |
| **Subcortical Volume** | | | | | | | | | |
|  | Thalamus Proper | -0.084 | -0.211 | 0.044 | 289548 | -0.082 | -0.224 | 0.061 | 235766 |
|  | Caudate | -0.088 | -0.22 | 0.044 | 238746 | -0.097 | -0.22 | 0.027 | 235759 |
|  | Putamen | -0.008 | -0.16 | 0.143 | 260101 | 0.074 | -0.072 | 0.217 | 251443 |
|  | Pallidum | 0.131 | -0.011 | 0.274 | 154010 | -0.008 | -0.163 | 0.143 | 283896 |
|  | Hippocampus | -0.244 | -0.399 | -0.087 | 276728 | -0.206 | -0.374 | -0.038 | 131309 |
|  | Amygdala | -0.171 | -0.313 | -0.03 | 279746 | -0.168 | -0.32 | -0.014 | 244304 |
|  | Nucleus Accumbens | -0.015 | -0.151 | 0.12 | 279471 | -0.123 | -0.27 | 0.026 | 272441 |
| **Surface Area** | | | | | | | | | |
|  | Banks Superior Temporal | -0.019 | -0.108 | 0.07 | 271582 | -0.036 | -0.147 | 0.073 | 246197 |
|  | Caudal Anterior Cingulate | -0.044 | -0.157 | 0.07 | 237090 | -0.006 | -0.144 | 0.134 | 261562 |
|  | Caudal Middle Frontal | -0.048 | -0.163 | 0.065 | 243702 | -0.059 | -0.183 | 0.063 | 245535 |
|  | Cuneus | 0.024 | -0.116 | 0.166 | 179713 | 0.039 | -0.104 | 0.183 | 152391 |
|  | Entorhinal | -0.033 | -0.14 | 0.073 | 257592 | -0.026 | -0.14 | 0.089 | 194874 |
|  | Fusiform | -0.118 | -0.265 | 0.033 | 151174 | -0.071 | -0.22 | 0.079 | 171898 |
|  | Inferior Parietal | -0.162 | -0.295 | -0.03 | 229334 | -0.091 | -0.195 | 0.013 | 248389 |
|  | Inferior Temporal | -0.093 | -0.263 | 0.08 | 187499 | -0.127 | -0.305 | 0.05 | 174941 |
|  | Isthmus Cingulate | -0.027 | -0.155 | 0.104 | 243075 | -0.045 | -0.191 | 0.102 | 221381 |
|  | Lateral Occipital | -0.056 | -0.187 | 0.073 | 248147 | -0.014 | -0.158 | 0.125 | 239889 |
|  | Lateral Orbitofrontal | -0.08 | -0.236 | 0.075 | 219036 | -0.095 | -0.23 | 0.043 | 187021 |
|  | Lingual | -0.088 | -0.201 | 0.025 | 235521 | -0.074 | -0.187 | 0.042 | 250447 |
|  | Medial Orbito Frontal | -0.043 | -0.167 | 0.08 | 260643 | -0.071 | -0.209 | 0.063 | 246253 |
|  | Middle Temporal | -0.042 | -0.191 | 0.106 | 292721 | -0.067 | -0.196 | 0.062 | 201346 |
|  | Parahippocampal | 0 | -0.148 | 0.149 | 95046 | -0.12 | -0.24 | 0.001 | 270810 |
|  | Para Central | -0.14 | -0.261 | -0.018 | 239352 | -0.086 | -0.194 | 0.023 | 245536 |
|  | Pars Opercularis | -0.095 | -0.217 | 0.03 | 176013 | -0.052 | -0.159 | 0.055 | 272986 |
|  | Pars Orbitalis | -0.066 | -0.216 | 0.086 | 138122 | -0.104 | -0.256 | 0.051 | 158353 |
|  | Pars Triangularis | -0.15 | -0.274 | -0.029 | 177237 | -0.02 | -0.161 | 0.121 | 265428 |
|  | Pericalcarine | -0.021 | -0.153 | 0.115 | 212868 | -0.028 | -0.17 | 0.117 | 155214 |
|  | Post Central | 0.051 | -0.081 | 0.186 | 209515 | -0.116 | -0.241 | 0.01 | 303391 |
|  | Posterior Cingulate | -0.02 | -0.151 | 0.112 | 278834 | -0.032 | -0.151 | 0.085 | 237609 |
|  | Pre Central | -0.092 | -0.213 | 0.031 | 241236 | -0.092 | -0.211 | 0.03 | 208366 |
|  | Precuneus | -0.049 | -0.226 | 0.13 | 157944 | -0.107 | -0.281 | 0.062 | 169768 |
|  | Rostral Anterior Cingulate | -0.048 | -0.178 | 0.081 | 196605 | 0.015 | -0.139 | 0.172 | 257623 |
|  | Rostral Middle Frontal | -0.109 | -0.247 | 0.03 | 226090 | -0.096 | -0.228 | 0.034 | 256685 |
|  | Superior Frontal | -0.098 | -0.224 | 0.028 | 256456 | -0.136 | -0.254 | -0.016 | 236520 |
|  | Superior Parietal | -0.137 | -0.271 | -0.001 | 219934 | -0.159 | -0.262 | -0.057 | 277658 |
|  | Superior Temporal | -0.098 | -0.211 | 0.016 | 255284 | -0.198 | -0.336 | -0.059 | 280535 |
|  | Supramarginal | -0.049 | -0.189 | 0.093 | 212228 | -0.096 | -0.23 | 0.037 | 271498 |
|  | Frontal Pole | -0.08 | -0.224 | 0.064 | 202046 | -0.075 | -0.188 | 0.04 | 193715 |
|  | Temporal Pole | 0.013 | -0.137 | 0.161 | 215546 | -0.041 | -0.176 | 0.096 | 274950 |
|  | Transverse Temporal | -0.134 | -0.254 | -0.015 | 221959 | -0.136 | -0.253 | -0.021 | 239247 |
|  | Insula | -0.009 | -0.138 | 0.121 | 185907 | -0.021 | -0.182 | 0.143 | 229902 |
| **Cortical Thickness** | | | | | | | | | |
|  | Banks Superior Temporal | -0.064 | -0.193 | 0.067 | 270084 | -0.108 | -0.236 | 0.022 | 240180 |
|  | Caudal Anterior Cingulate | -0.103 | -0.223 | 0.017 | 213993 | -0.001 | -0.136 | 0.134 | 186679 |
|  | Caudal Middle Frontal | -0.211 | -0.333 | -0.087 | 203579 | -0.172 | -0.316 | -0.026 | 197976 |
|  | Cuneus | 0.008 | -0.146 | 0.161 | 214098 | 0.013 | -0.127 | 0.152 | 195869 |
|  | Entorhinal | -0.029 | -0.178 | 0.121 | 210657 | 0.005 | -0.137 | 0.147 | 150918 |
|  | Fusiform | -0.108 | -0.227 | 0.013 | 226865 | -0.149 | -0.306 | 0.007 | 146331 |
|  | Inferior Parietal | -0.097 | -0.232 | 0.04 | 253543 | -0.029 | -0.15 | 0.09 | 223978 |
|  | Inferior Temporal | -0.031 | -0.163 | 0.099 | 184186 | -0.065 | -0.214 | 0.084 | 87105 |
|  | Isthmus Cingulate | -0.139 | -0.274 | -0.003 | 220582 | -0.132 | -0.26 | -0.002 | 206525 |
|  | Lateral Occipital | -0.069 | -0.192 | 0.052 | 267104 | -0.062 | -0.193 | 0.069 | 211726 |
|  | Lateral Orbitofrontal | -0.092 | -0.224 | 0.039 | 183576 | -0.182 | -0.315 | -0.049 | 245835 |
|  | Lingual | -0.056 | -0.21 | 0.101 | 265564 | -0.036 | -0.161 | 0.091 | 142103 |
|  | Medial Orbito Frontal | -0.169 | -0.305 | -0.03 | 205651 | -0.088 | -0.239 | 0.059 | 197851 |
|  | Middle Temporal | -0.021 | -0.151 | 0.114 | 235489 | -0.055 | -0.178 | 0.067 | 160199 |
|  | Parahippocampal | -0.112 | -0.25 | 0.027 | 277469 | -0.115 | -0.243 | 0.014 | 256835 |
|  | Para Central | -0.109 | -0.268 | 0.054 | 250415 | -0.045 | -0.168 | 0.08 | 301501 |
|  | Pars Opercularis | -0.097 | -0.228 | 0.033 | 261187 | -0.064 | -0.19 | 0.061 | 273916 |
|  | Pars Orbitalis | -0.077 | -0.195 | 0.039 | 287464 | -0.076 | -0.196 | 0.046 | 240389 |
|  | Pars Triangularis | -0.085 | -0.221 | 0.05 | 247089 | -0.086 | -0.223 | 0.051 | 296476 |
|  | Pericalcarine | -0.018 | -0.174 | 0.144 | 260866 | -0.018 | -0.195 | 0.157 | 237123 |
|  | Post Central | -0.006 | -0.163 | 0.153 | 315881 | -0.047 | -0.212 | 0.12 | 302897 |
|  | Posterior Cingulate | -0.166 | -0.298 | -0.034 | 234894 | -0.097 | -0.24 | 0.045 | 161092 |
|  | Pre Central | -0.137 | -0.292 | 0.015 | 189552 | -0.152 | -0.294 | -0.007 | 275094 |
|  | Precuneus | -0.085 | -0.221 | 0.049 | 253552 | -0.019 | -0.157 | 0.122 | 170681 |
|  | Rostral Anterior Cingulate | -0.16 | -0.297 | -0.022 | 200339 | -0.153 | -0.298 | -0.011 | 193377 |
|  | Rostral Middle Frontal | -0.089 | -0.229 | 0.049 | 246913 | -0.074 | -0.214 | 0.066 | 275559 |
|  | Superior Frontal | -0.181 | -0.292 | -0.069 | 241411 | -0.149 | -0.261 | -0.041 | 237091 |
|  | Superior Parietal | -0.08 | -0.218 | 0.058 | 295567 | -0.015 | -0.148 | 0.118 | 257342 |
|  | Superior Temporal | -0.094 | -0.248 | 0.06 | 310264 | -0.092 | -0.222 | 0.034 | 294732 |
|  | Supramarginal | -0.097 | -0.233 | 0.038 | 296491 | -0.112 | -0.253 | 0.031 | 267709 |
|  | Frontal Pole | -0.042 | -0.173 | 0.088 | 179355 | -0.032 | -0.148 | 0.085 | 238430 |
|  | Temporal Pole | -0.095 | -0.213 | 0.02 | 256819 | -0.147 | -0.255 | -0.038 | 250369 |
|  | Transverse Temporal | -0.05 | -0.183 | 0.084 | 273498 | 0.006 | -0.099 | 0.115 | 245088 |
|  | Insula | -0.187 | -0.333 | -0.042 | 134469 | -0.177 | -0.305 | -0.05 | 219328 |

**Table S3.** Results of the one-sided one-sample t-test on the magnitude of adjustment between the original and Bayesian adjusted (i.e., posterior mode) effect sizes for each study.

| Study ID | Estimate | Statistic | p.value | df | Sample size |
| --- | --- | --- | --- | --- | --- |
| study_1 | 0.06354 | 8.206099 | 4.96E-14 | 149 | 27 |
| study_2 | 0.05332 | 8.763419 | 1.96E-15 | 149 | 30 |
| study_3 | 0.062693 | 7.37872 | 5.19E-12 | 149 | 30 |
| study_4 | 0.04168 | 13.18385 | 4.28E-27 | 149 | 34 |
| study_5 | 0.026131 | 14.00247 | 2.90E-29 | 149 | 39 |
| study_6 | 0.029306 | 12.39304 | 5.46E-25 | 149 | 40 |
| study_7 | 0.028986 | 13.17904 | 4.40E-27 | 149 | 41 |
| study_8 | 0.038605 | 12.72628 | 7.06E-26 | 149 | 41 |
| study_9 | 0.024281 | 19.22517 | 1.71E-42 | 149 | 52 |
| study_10 | 0.015115 | 16.50291 | 9.41E-36 | 149 | 59 |
| study_11 | 0.016864 | 18.82041 | 1.62E-41 | 149 | 66 |
| study_12 | 0.012869 | 17.26132 | 1.14E-37 | 149 | 69 |
| study_13 | 0.012479 | 17.15957 | 2.06E-37 | 149 | 71 |
| study_14 | 0.014145 | 17.31293 | 8.49E-38 | 149 | 78 |
| study_15 | 0.010609 | 18.53369 | 8.11E-41 | 149 | 91 |
| study_16 | 0.00683 | 15.41485 | 5.85E-33 | 149 | 102 |
| study_17 | 0.013465 | 22.12475 | 3.08E-49 | 149 | 111 |
| study_18 | 0.005317 | 16.39356 | 1.79E-35 | 149 | 116 |
| study_19 | 0.004877 | 16.38776 | 1.85E-35 | 149 | 136 |
| study_20 | 0.00229 | 19.62636 | 1.87E-43 | 149 | 298 |
| study_21 | 0.001449 | 15.35192 | 8.52E-33 | 149 | 368 |

**Table S4.** Results of the one-sided one-sample t-test on the magnitude of adjustment between the original and Bayesian adjusted (i.e., posterior mean) effect sizes for each study.

| Study ID | Estimate | Statistic | p.value | df | Sample size |
| --- | --- | --- | --- | --- | --- |
| study_1 | 0.114797 | 13.05495 | 9.41E-27 | 149 | 27 |
| study_2 | 0.108134 | 11.9017 | 1.12E-23 | 149 | 30 |
| study_3 | 0.125568 | 12.43521 | 4.21E-25 | 149 | 30 |
| study_4 | 0.086971 | 15.22929 | 1.77E-32 | 149 | 34 |
| study_5 | 0.058523 | 14.08911 | 1.71E-29 | 149 | 39 |
| study_6 | 0.078137 | 10.52769 | 5.04E-20 | 149 | 40 |
| study_7 | 0.067592 | 15.08866 | 4.12E-32 | 149 | 41 |
| study_8 | 0.084463 | 13.60719 | 3.22E-28 | 149 | 41 |
| study_9 | 0.066149 | 17.12345 | 2.54E-37 | 149 | 52 |
| study_10 | 0.040817 | 15.51917 | 3.14E-33 | 149 | 59 |
| study_11 | 0.046028 | 15.93799 | 2.62E-34 | 149 | 66 |
| study_12 | 0.037407 | 14.1473 | 1.20E-29 | 149 | 69 |
| study_13 | 0.037231 | 14.28959 | 5.08E-30 | 149 | 71 |
| study_14 | 0.042422 | 15.80043 | 5.91E-34 | 149 | 78 |
| study_15 | 0.033205 | 15.67779 | 1.22E-33 | 149 | 91 |
| study_16 | 0.020621 | 13.81164 | 9.26E-29 | 149 | 102 |
| study_17 | 0.044142 | 18.34232 | 2.38E-40 | 149 | 111 |
| study_18 | 0.018226 | 17.06012 | 3.66E-37 | 149 | 116 |
| study_19 | 0.01534 | 15.13867 | 3.05E-32 | 149 | 136 |
| study_20 | 0.008712 | 17.29145 | 9.61E-38 | 149 | 298 |
| study_21 | 0.004629 | 13.11083 | 6.69E-27 | 149 | 368 |

## Supplementary Figures

**Figure S1.** Posterior distributions of the overarching parameter M for surface area. **A.** Posterior mode for each region mapped onto the brain. **B.** The posterior distribution for cortical regions. The dashed line indicates the posterior mode and the light blue area denotes the 95% highest density interval (HDI). Regions are sorted by the mode value of the distribution.


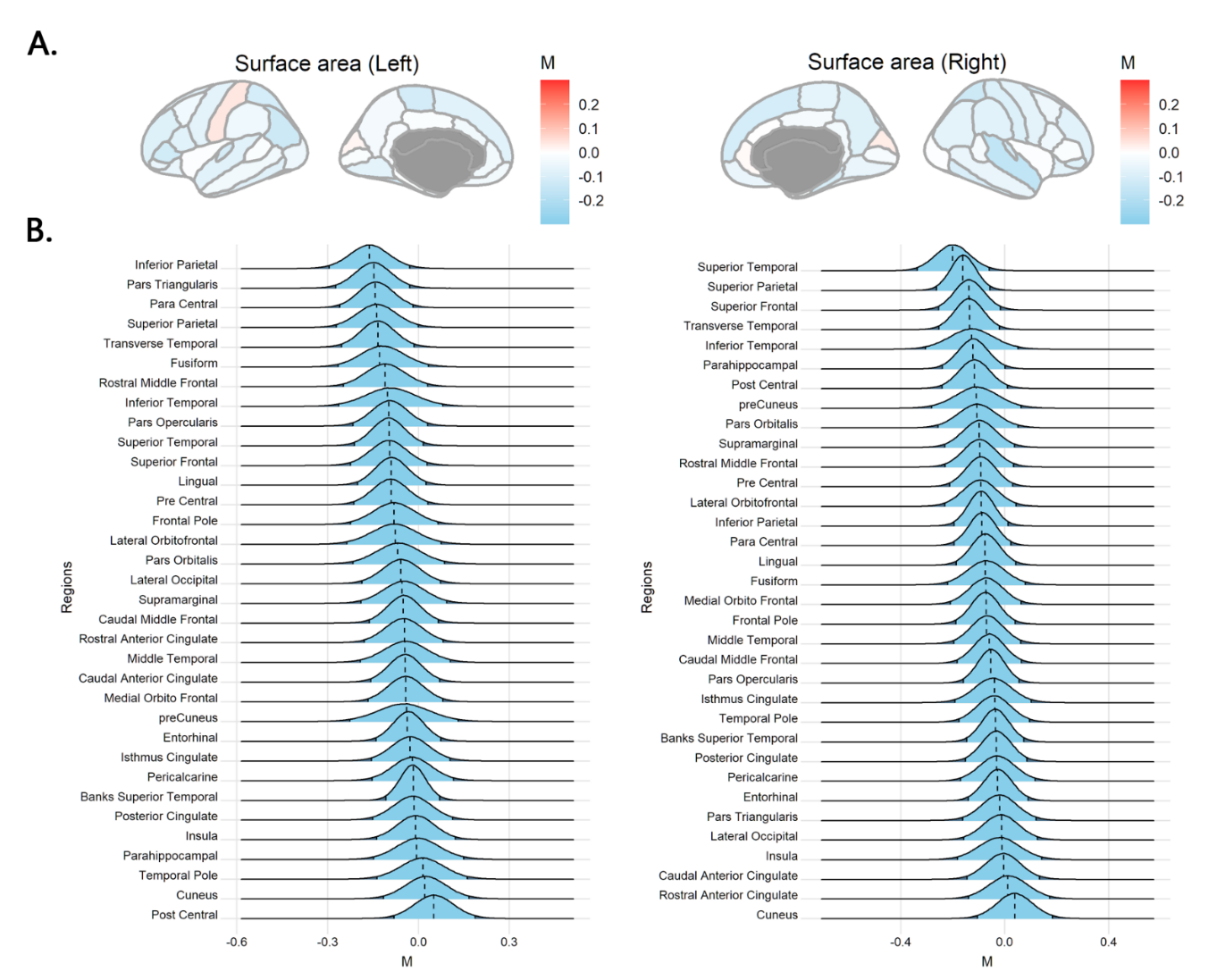


**Figure S2.** Posterior distributions of the overarching parameter *M* for subcortical volume. **A.** Posterior mode for each region mapped onto the brain. **B.** The posterior distribution for subcortical regions. The dashed line indicates the posterior mode and the light blue area denotes the 95% highest density interval (HDI). Regions are sorted by the mode value of the distribution.


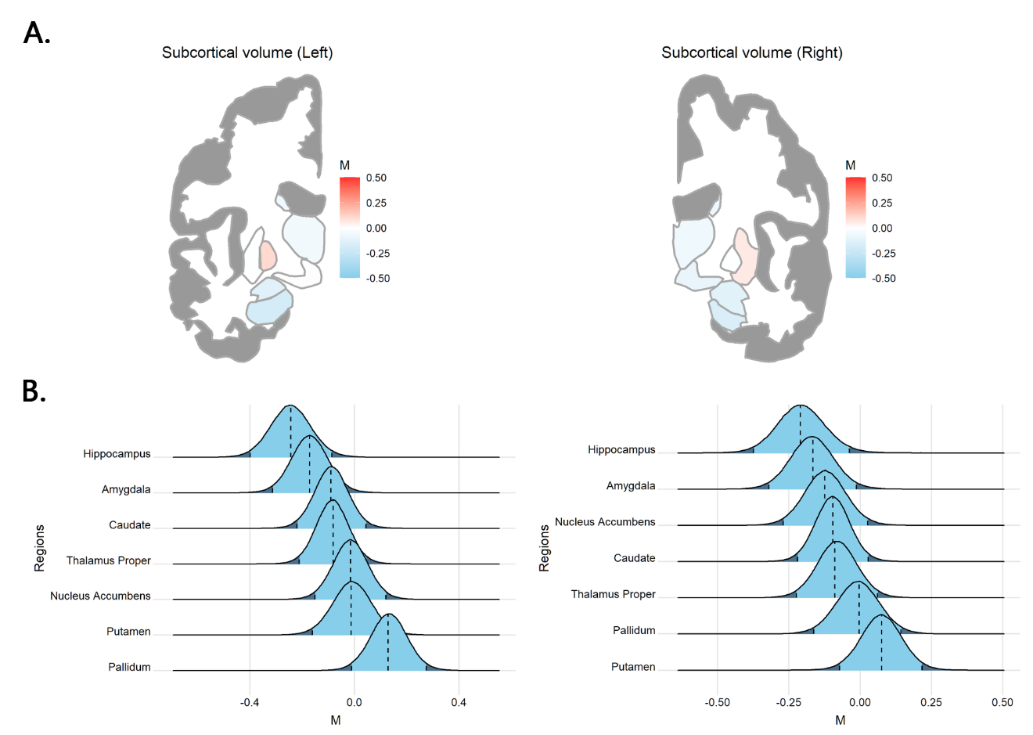


**Figure S3. A.** The original effect size (blue) and Bayesian adjusted effect size (red; i.e., the posterior mode of the study-specific parameter *µ*) for the cortical thickness of the left caudal middle frontal cortex and right lateral orbitofrontal cortex. The horizontal arrow indicates the adjustment from the original effect size toward the Bayesian adjusted effect size. The dashed line and the grey shaded area denote the posterior mode and the 95% HDI of the overarching parameter *M* respectively. **B.** The original effect size (blue) and Bayesian adjusted effect size (red; i.e., the posterior mean of the study-specific parameter *µ*) for the cortical thickness of the left caudal middle frontal cortex and right lateral orbitofrontal cortex.


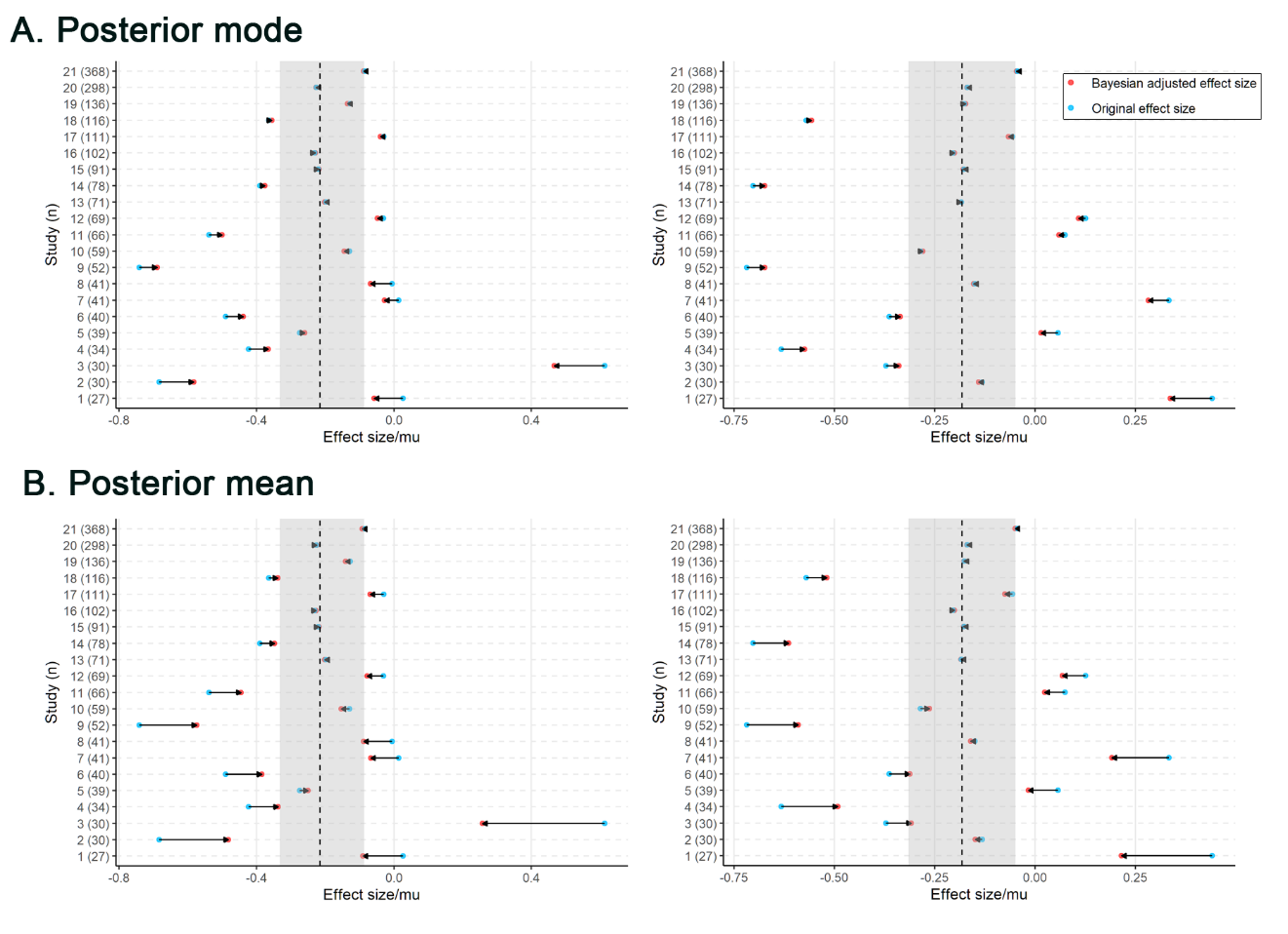


**Figure S4. A.** The posterior distribution of the study-specific parameter *µ* and the overarching parameter *M* (top line) for the surface area of the left parahippocampal gyrus and right cuneus gyrus. The dashed line indicates the posterior mode of the parameters and the light blue, as well as the grey shaded area, denotes the 95% highest density interval (HDI). **B.** The original effect size (blue) and Bayesian adjusted effect size (red; i.e., the posterior mode of the study-specific parameter *µ*) for the surface area of the left parahippocampal gyrus and right cuneus gyrus. The horizontal arrow indicates the adjustment from the original effect size toward the Bayesian adjusted effect size. The dashed line and the grey shaded area denote the posterior mode and the 95% HDI of the overarching parameter *M* respectively.

**
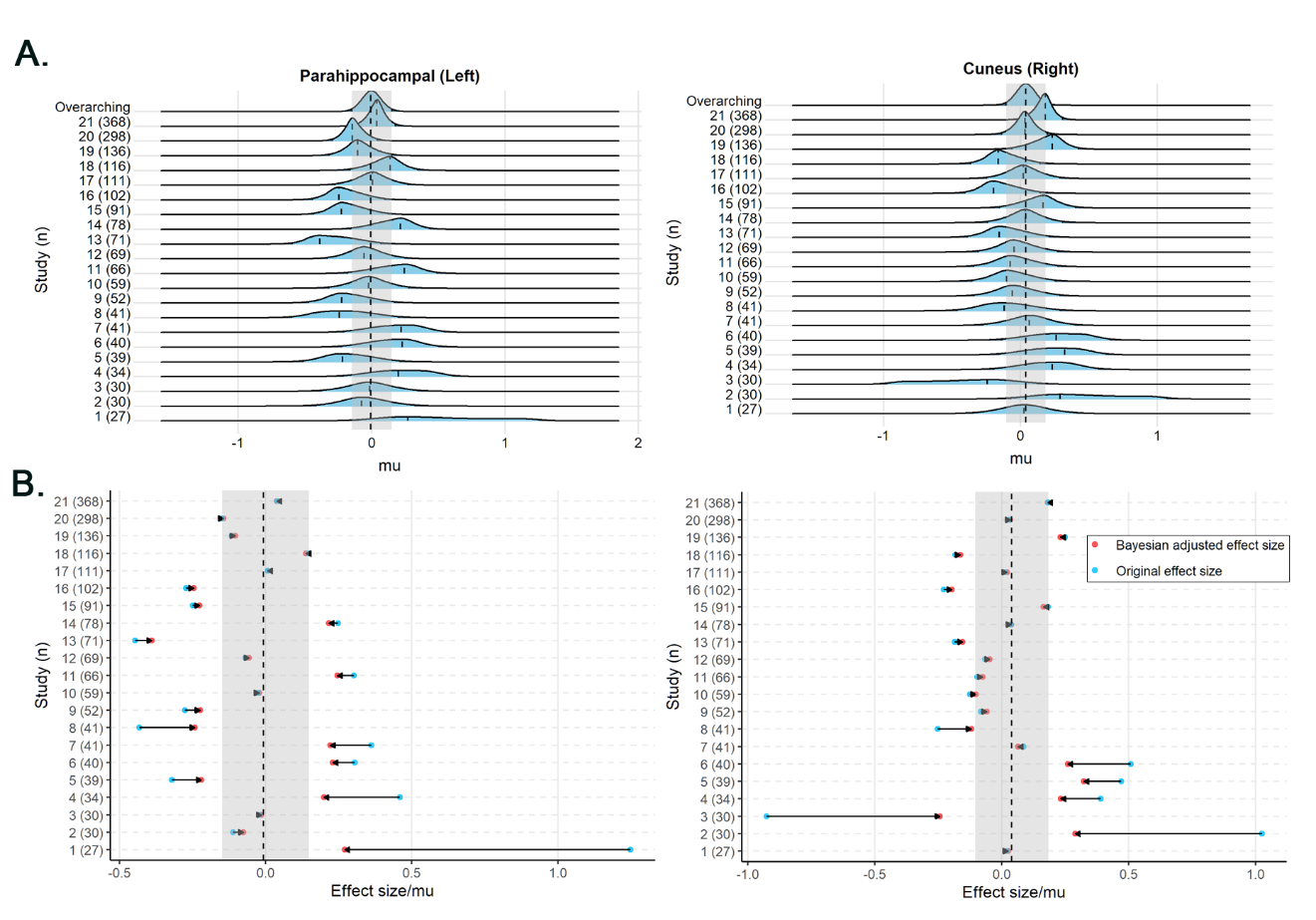
**

**Figure S5.** Violin plot of p-values from the Kolmogorov-Smirnov (KS) normality tests for 1,000 simulated effect sizes across 21 sites. Each violin corresponds to one site, with the sample size indicated in parentheses. The dots represent individual p-values for each regional measurement. The horizontal red dashed line denotes the p-value threshold of 0.05, below which the null hypothesis (i.e., the simulated effect sizes are drawn from a normal distribution) is rejected.

**
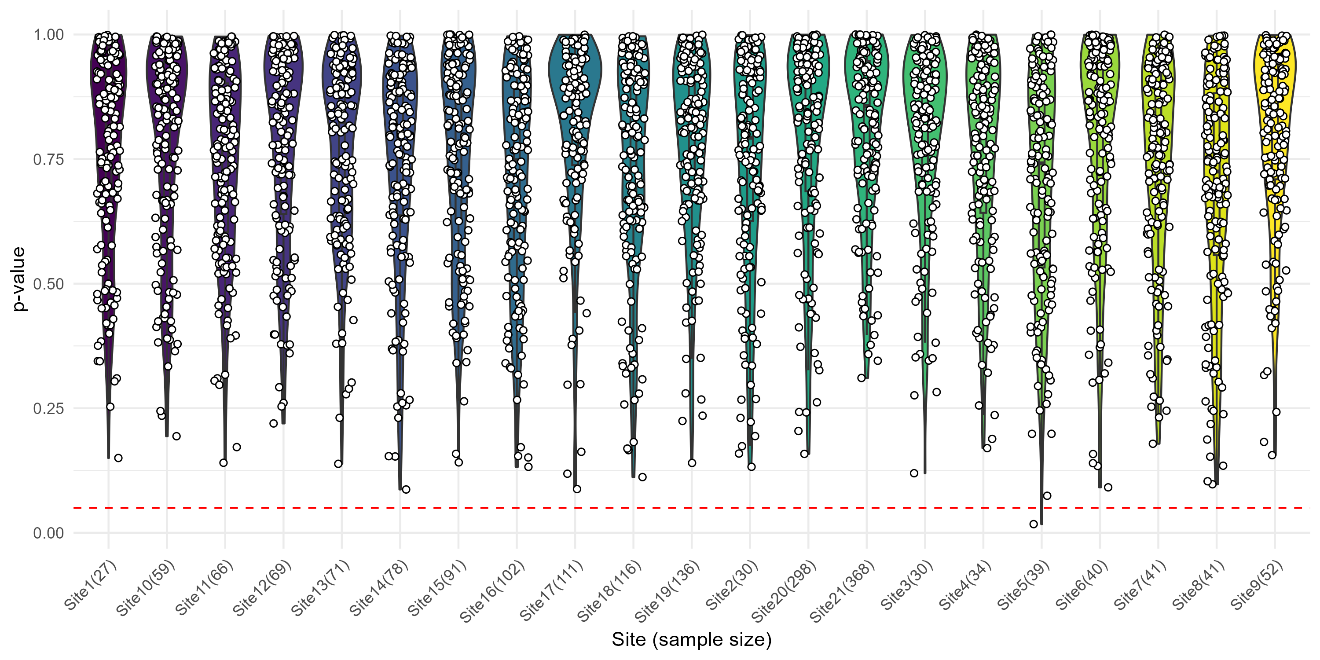
**

**Figure S6**. Posterior distributions of the overarching parameter *M* for cortical thickness estimated using data without Combat harmonization. A. Posterior mode for each region mapped onto the brain. B. The posterior distribution for cortical regions. The dashed line indicates the posterior mode and the light blue area denotes the 95% highest density interval (HDI). Regions are sorted by the mode value of the distribution.


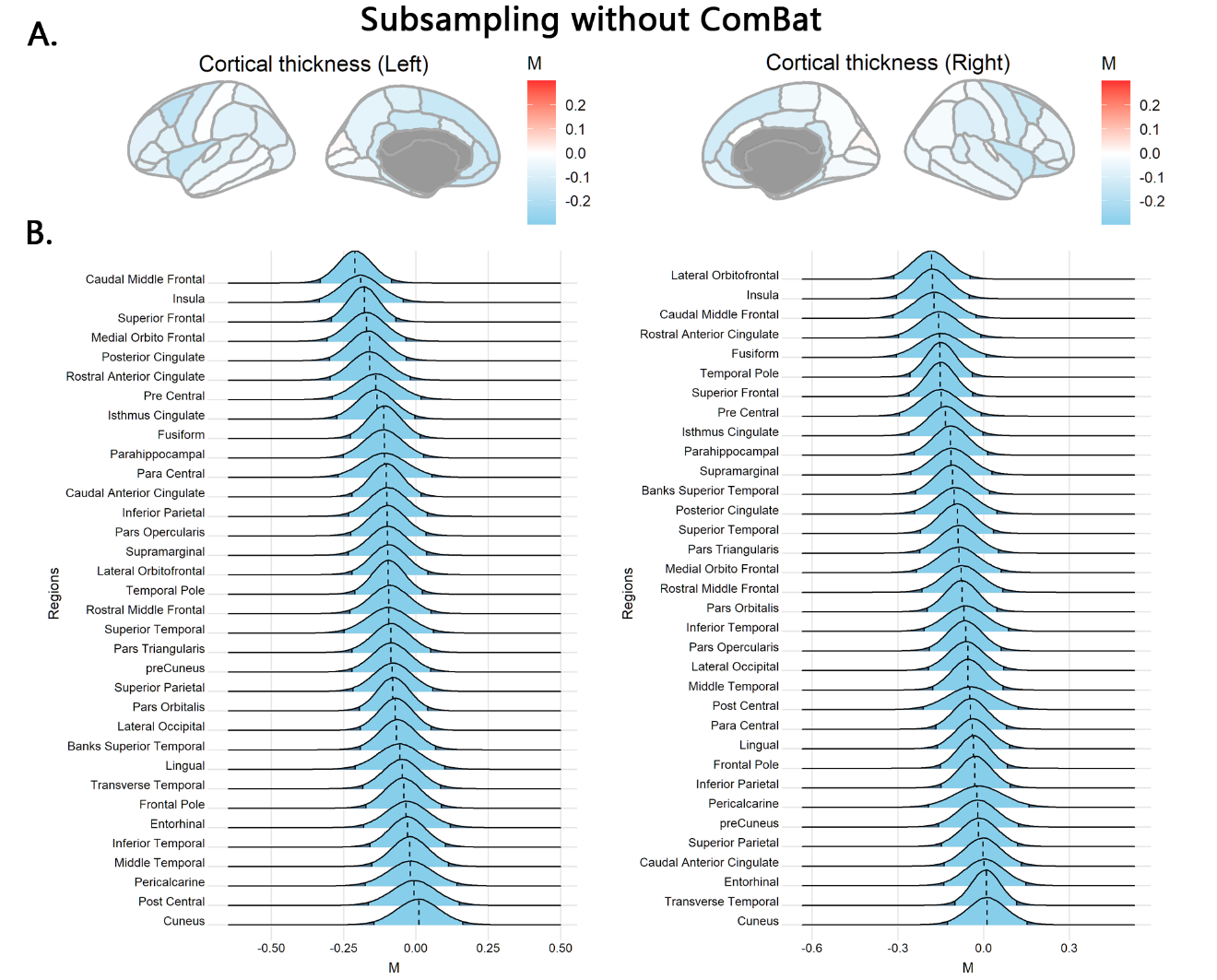


**Figure S7**. Results of overarching and site-specific estimates using data without ComBat harmonization. **A.** The posterior distribution of the study-specific parameter µ and the overarching parameter *M* (top line) for the cortical thickness of the left caudal middle frontal cortex and right lateral orbitofrontal cortex. The dashed line indicates the posterior mode of the parameters and the light blue, as well as the grey shaded area, denotes the 95% highest density interval (HDI). B. The original effect size (blue) and Bayesian adjusted effect size (red; i.e., the posterior mode of the study-specific parameter *µ*) for the cortical thickness of the left caudal middle frontal cortex and right lateral orbitofrontal cortex. The horizontal arrow indicates the adjustment from the original effect size toward the Bayesian adjusted effect size. The dashed line and the grey shaded area denote the posterior mode and the 95% HDI of the overarching parameter *M* respectively.


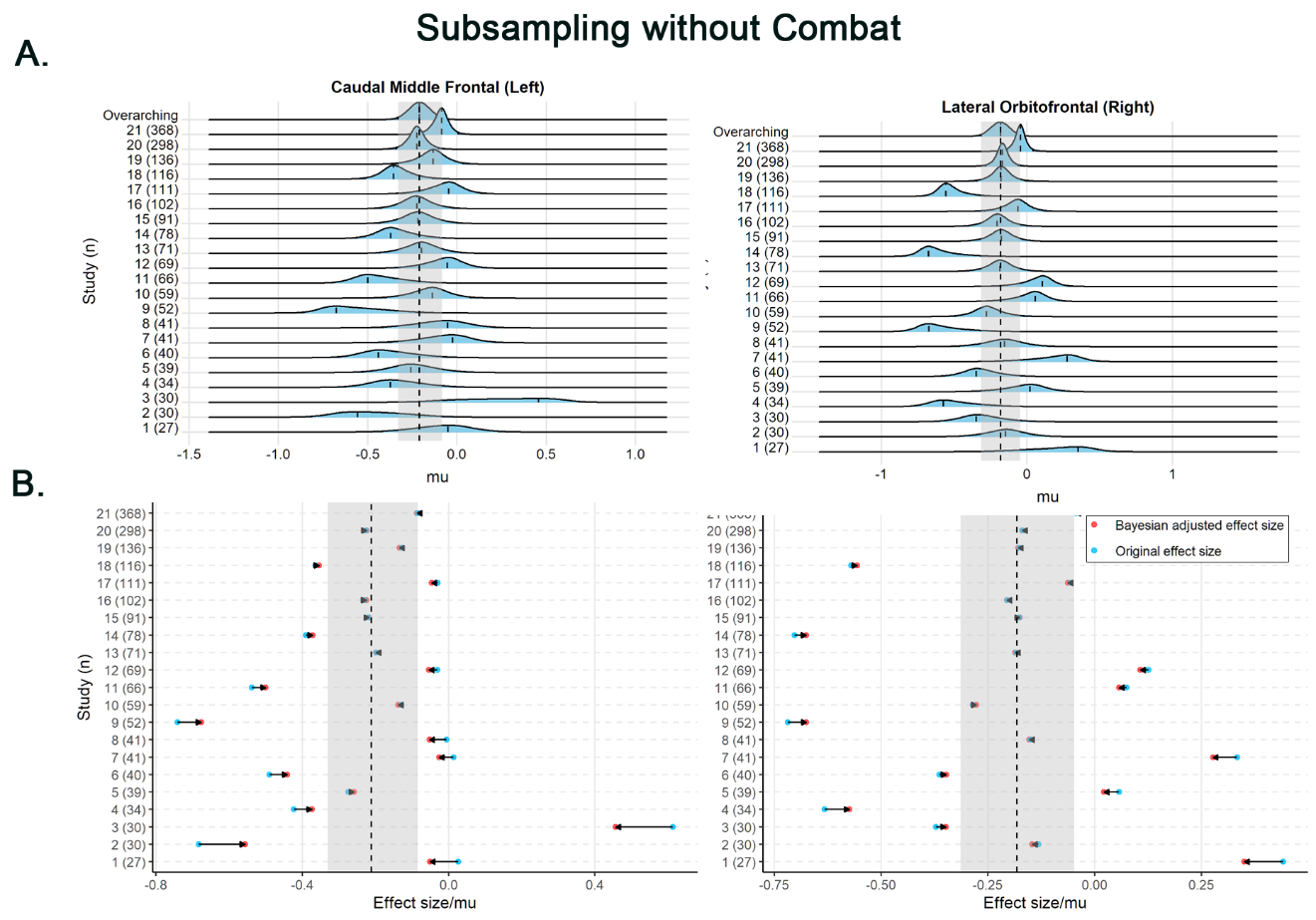


**Figure S8.** Gamma priors with different parameters. The left panel shows the mildly informative gamma prior used in the main analysis with a mode of 1 and an SD of 10. The middle panel shows the Gamma prior with a mode of 1 and an SD of 100. This prior may not be a reasonable choice for the variance of effect sizes that typically range between -1 to 1. The right panel shows the Gamma prior with a mode of 1 and an SD of 0.1. This prior is not typical for practical use because the standard deviation is small, resulting in a distribution that closely resembles a delta at the mode.


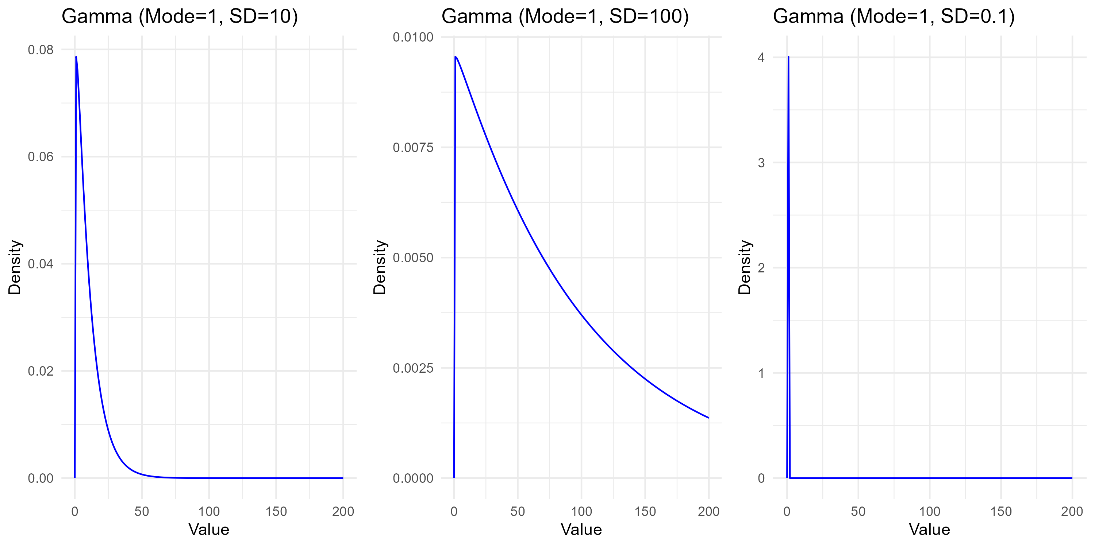


**Figure S9.** Posterior distributions of the overarching parameter M for cortical thickness estimated using the Gamma prior with a mode of 1 and an SD of 0.1. A. Posterior mode for each region mapped onto the brain. B. The posterior distribution for cortical regions. The dashed line indicates the posterior mode and the light blue area denotes the 95% highest density interval (HDI). Regions are sorted by the mode value of the distribution.

**
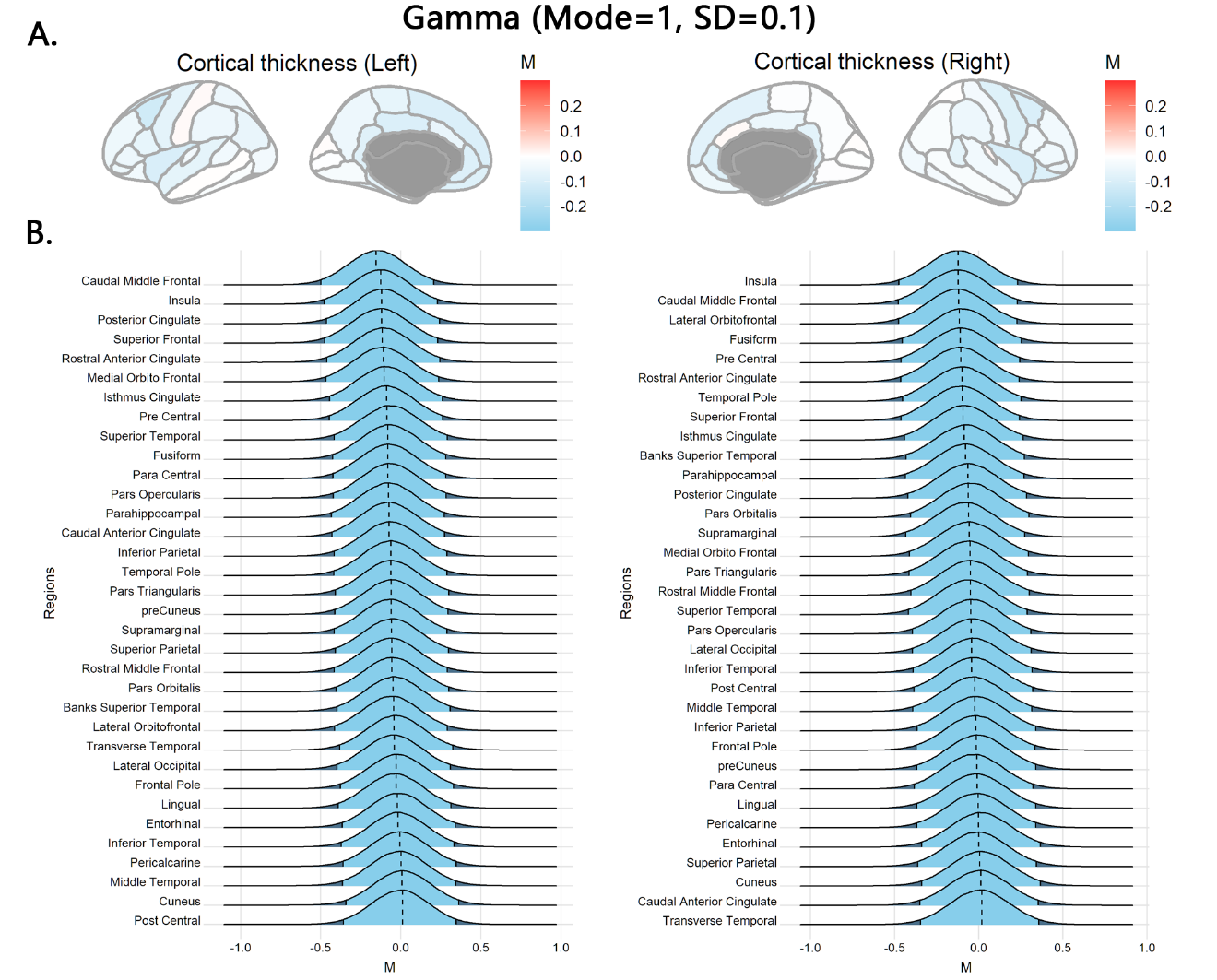
**

**Figure S10**. Results of overarching and site-specific estimates using the Gamma prior with a mode of 1 and an SD of 0.1. **A.** The posterior distribution of the study-specific parameter µ and the overarching parameter *M* (top line) for the cortical thickness of the left caudal middle frontal cortex and right lateral orbitofrontal cortex. The dashed line indicates the posterior mode of the parameters and the light blue, as well as the grey shaded area, denotes the 95% highest density interval (HDI). B. The original effect size (blue) and Bayesian adjusted effect size (red; i.e., the posterior mode of the study-specific parameter *µ*) for the cortical thickness of the left caudal middle frontal cortex and right lateral orbitofrontal cortex. The horizontal arrow indicates the adjustment from the original effect size toward the Bayesian adjusted effect size. The dashed line and the grey shaded area denote the posterior mode and the 95% HDI of the overarching parameter *M* respectively.

**
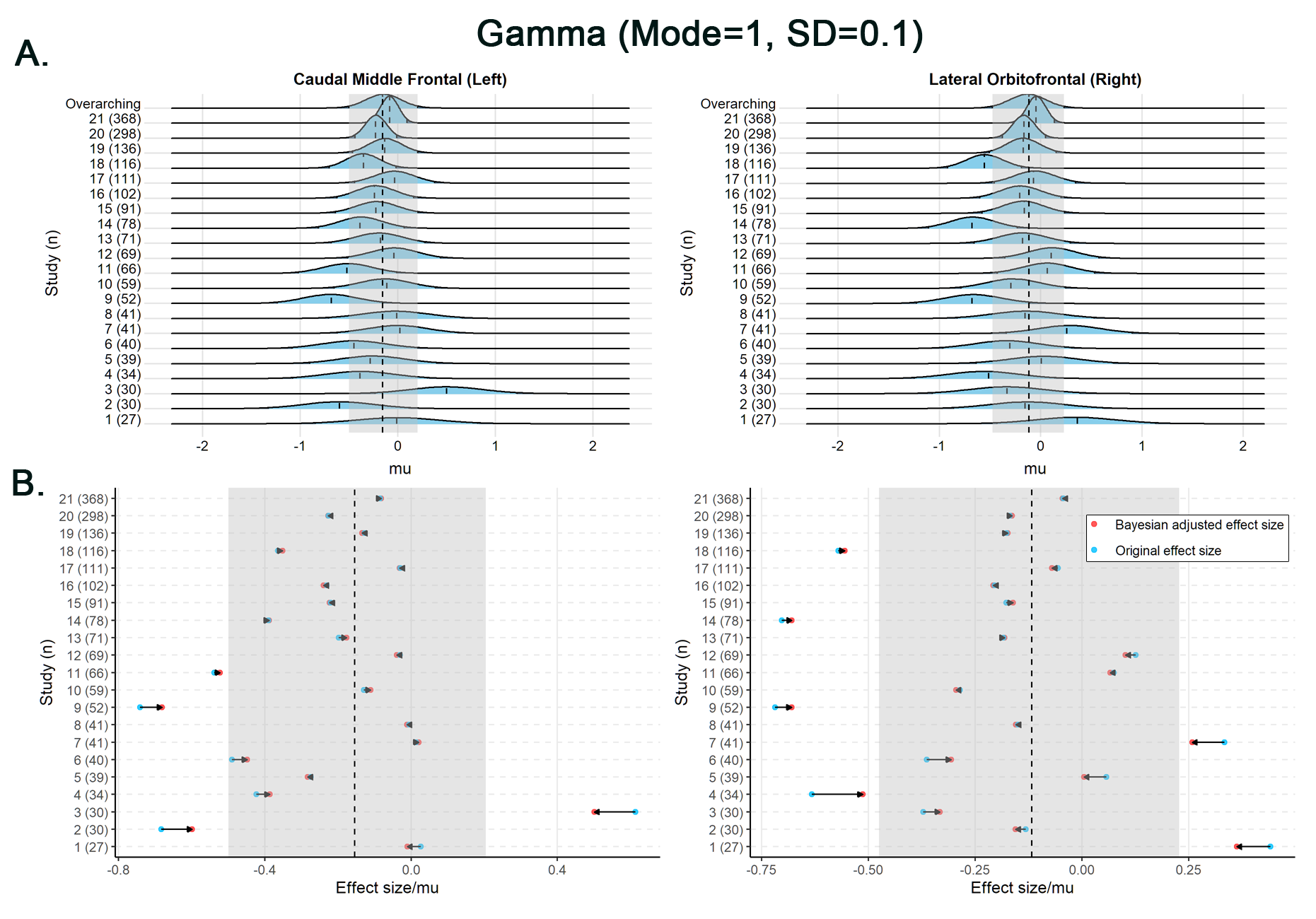
**

**Figure S11.** Posterior distributions of the overarching parameter M for cortical thickness estimated using the Gamma prior with a mode of 1 and an SD of 100. **A.** Posterior mode for each region mapped onto the brain. **B.** The posterior distribution for cortical regions. The dashed line indicates the posterior mode and the light blue area denotes the 95% highest density interval (HDI). Regions are sorted by the mode value of the distribution.

**
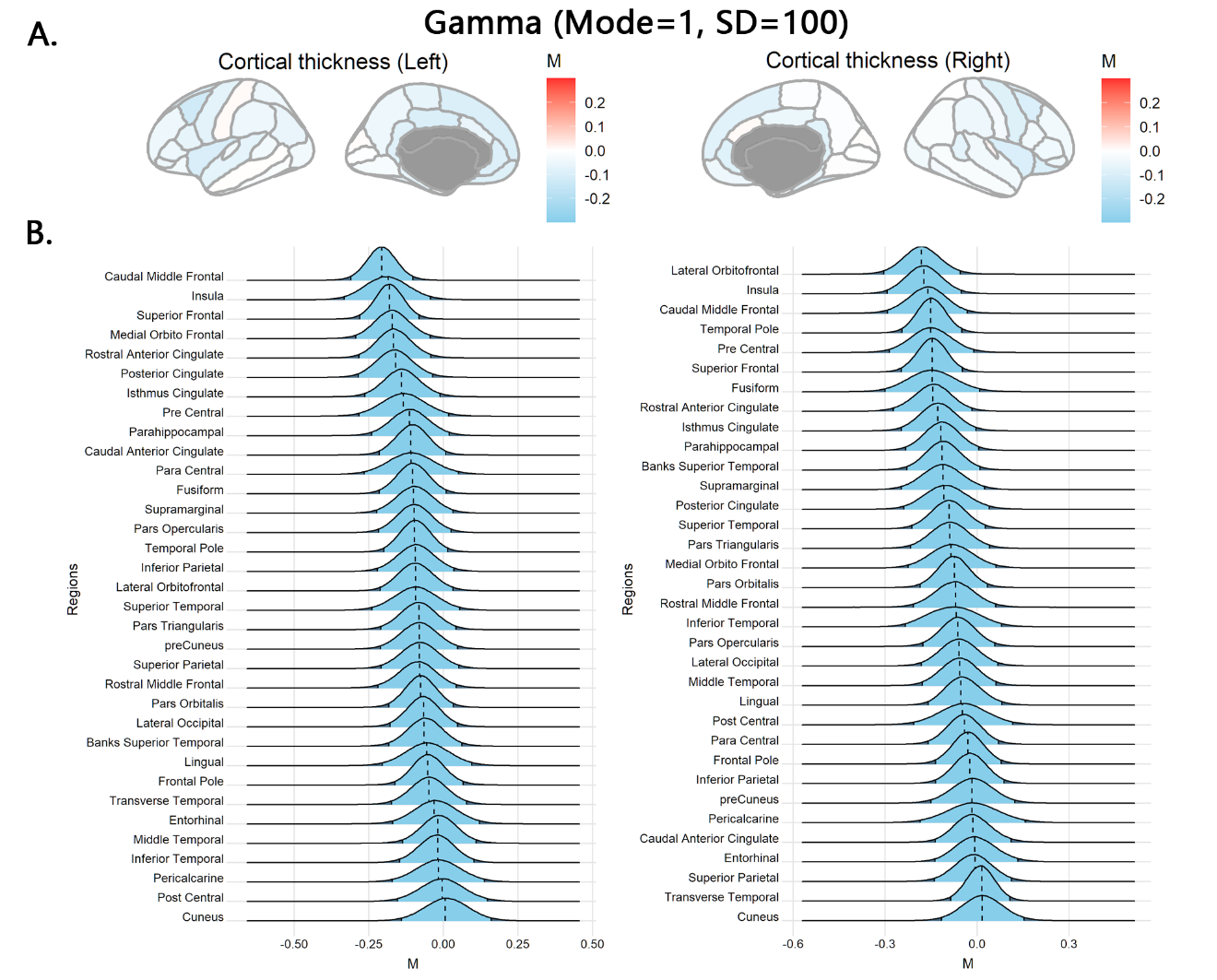
**

**Figure S12**. Results of overarching and site-specific estimates using the Gamma prior with a mode of 1 and an SD of 100. **A.** The posterior distribution of the study-specific parameter µ and the overarching parameter *M* (top line) for the cortical thickness of the left caudal middle frontal cortex and right lateral orbitofrontal cortex. The dashed line indicates the posterior mode of the parameters and the light blue, as well as the grey shaded area, denotes the 95% highest density interval (HDI). B. The original effect size (blue) and Bayesian adjusted effect size (red; i.e., the posterior mode of the study-specific parameter *µ*) for the cortical thickness of the left caudal middle frontal cortex and right lateral orbitofrontal cortex. The horizontal arrow indicates the adjustment from the original effect size toward the Bayesian adjusted effect size. The dashed line and the grey shaded area denote the posterior mode and the 95% HDI of the overarching parameter *M* respectively.

**
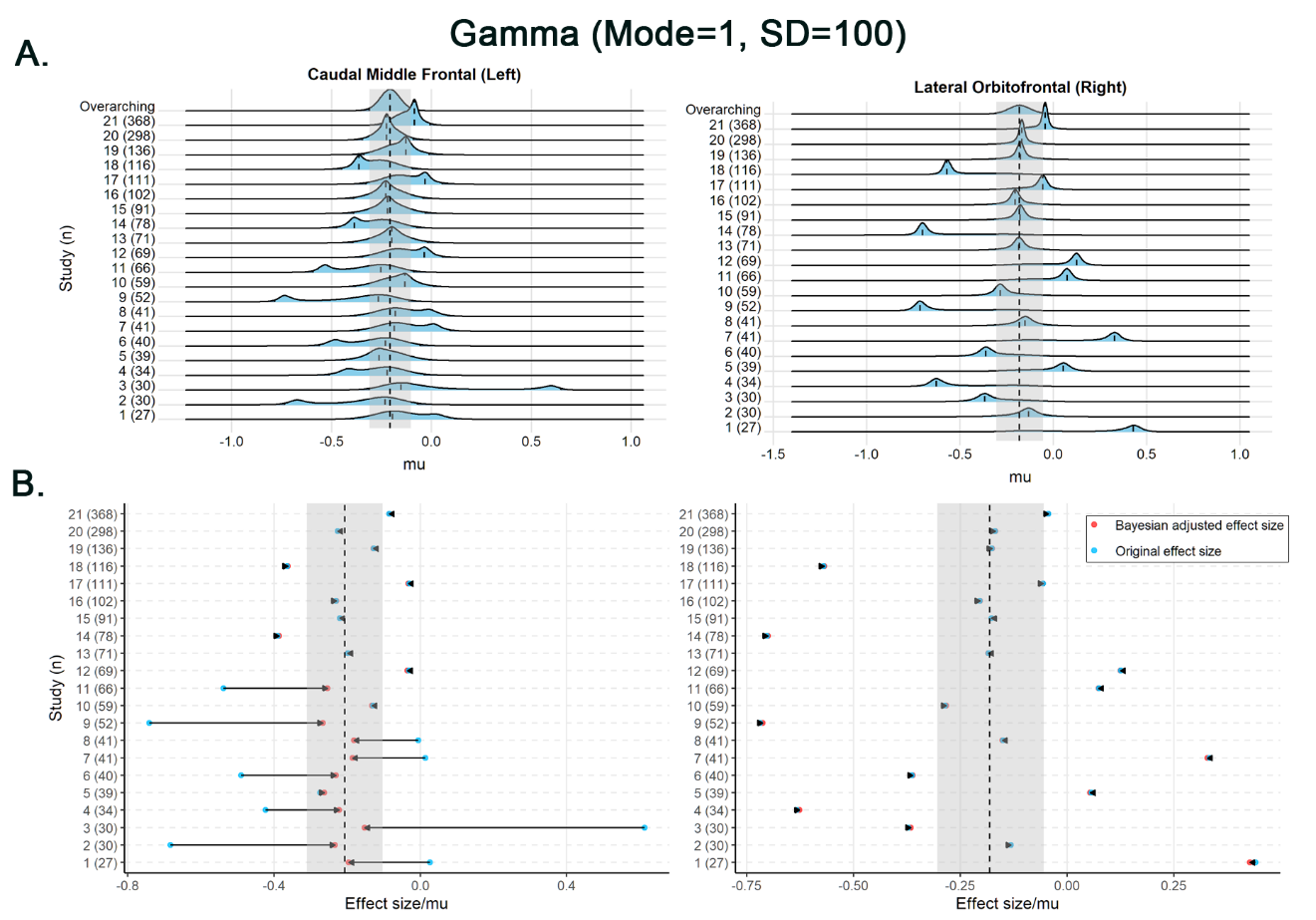
**

## References

Boedhoe, P.S., Schmaal, L., Abe, Y., Alonso, P., Ameis, S.H., Anticevic, A., Arnold, P.D., Batistuzzo, M.C., Benedetti, F., Beucke, J.C. (2018) Cortical abnormalities associated with pediatric and adult obsessive-compulsive disorder: findings from the ENIGMA Obsessive-Compulsive Disorder Working Group. American Journal of Psychiatry, 175:453-462.

Cao, Z., Cupertino, R.B., Ottino-Gonzalez, J., Murphy, A., Pancholi, D., Juliano, A., Chaarani, B., Albaugh, M., Yuan, D., Schwab, N. (2023) Cortical profiles of numerous psychiatric disorders and normal development share a common pattern. Molecular Psychiatry, 28:698-709.

Cao, Z., Ottino‐Gonzalez, J., Cupertino, R.B., Schwab, N., Hoke, C., Catherine, O., Cousijn, J., Dagher, A., Foxe, J.J., Goudriaan, A.E. (2021) Mapping cortical and subcortical asymmetries in substance dependence: Findings from the ENIGMA Addiction Working Group. Addiction Biology:e13010.

Kruschke, J. (2014) Doing Bayesian data analysis: A tutorial with R, JAGS, and Stan. Academic Press.

Schmaal, L., Hibar, D., Sämann, P.G., Hall, G., Baune, B., Jahanshad, N., Cheung, J., van Erp, T.G., Bos, D., Ikram, M.A. (2017) Cortical abnormalities in adults and adolescents with major depression based on brain scans from 20 cohorts worldwide in the ENIGMA Major Depressive Disorder Working Group. Molecular Psychiatry, 22:900-909.

Van Erp, T.G., Walton, E., Hibar, D.P., Schmaal, L., Jiang, W., Glahn, D.C., Pearlson, G.D., Yao, N., Fukunaga, M., Hashimoto, R. (2018) Cortical brain abnormalities in 4474 individuals with schizophrenia and 5098 control subjects via the enhancing neuro imaging genetics through meta analysis (ENIGMA) consortium. Biological Psychiatry, 84:644-654.

Whelan, C.D., Altmann, A., Botía, J.A., Jahanshad, N., Hibar, D.P., Absil, J., Alhusaini, S., Alvim, M.K., Auvinen, P., Bartolini, E. (2018) Structural brain abnormalities in the common epilepsies assessed in a worldwide ENIGMA study. Brain, 141:391-408.
